# The fractal brain: scale-invariance in structure and dynamics

**DOI:** 10.1093/cercor/bhac363

**Published:** 2022-09-26

**Authors:** George F Grosu, Alexander V Hopp, Vasile V Moca, Harald Bârzan, Andrei Ciuparu, Maria Ercsey-Ravasz, Mathias Winkel, Helmut Linde, Raul C Mureșan

**Affiliations:** Department of Experimental and Theoretical Neuroscience, Transylvanian Institute of Neuroscience, Str. Ploiesti 33, 400157 Cluj-Napoca, Romania; Faculty of Electronics, Telecommunications and Information Technology, Technical University of Cluj-Napoca, Str. Memorandumului 28, 400114 Cluj-Napoca, Romania; Merck KGaA, Frankfurter Straße 250, 64293 Darmstadt, Germany; Department of Experimental and Theoretical Neuroscience, Transylvanian Institute of Neuroscience, Str. Ploiesti 33, 400157 Cluj-Napoca, Romania; Department of Experimental and Theoretical Neuroscience, Transylvanian Institute of Neuroscience, Str. Ploiesti 33, 400157 Cluj-Napoca, Romania; Faculty of Electronics, Telecommunications and Information Technology, Technical University of Cluj-Napoca, Str. Memorandumului 28, 400114 Cluj-Napoca, Romania; Department of Experimental and Theoretical Neuroscience, Transylvanian Institute of Neuroscience, Str. Ploiesti 33, 400157 Cluj-Napoca, Romania; Faculty of Electronics, Telecommunications and Information Technology, Technical University of Cluj-Napoca, Str. Memorandumului 28, 400114 Cluj-Napoca, Romania; Department of Experimental and Theoretical Neuroscience, Transylvanian Institute of Neuroscience, Str. Ploiesti 33, 400157 Cluj-Napoca, Romania; Faculty of Physics, Babes-Bolyai University, Str. Mihail Kogalniceanu 1, 400084 Cluj-Napoca, Romania; Merck KGaA, Frankfurter Straße 250, 64293 Darmstadt, Germany; Department of Experimental and Theoretical Neuroscience, Transylvanian Institute of Neuroscience, Str. Ploiesti 33, 400157 Cluj-Napoca, Romania; Merck KGaA, Frankfurter Straße 250, 64293 Darmstadt, Germany; Department of Experimental and Theoretical Neuroscience, Transylvanian Institute of Neuroscience, Str. Ploiesti 33, 400157 Cluj-Napoca, Romania

**Keywords:** fractality, self-organized criticality, scale-free structure, scale-free dynamics, power law

## Abstract

The past 40 years have witnessed extensive research on fractal structure and scale-free dynamics in the brain. Although considerable progress has been made, a comprehensive picture has yet to emerge, and needs further linking to a mechanistic account of brain function. Here, we review these concepts, connecting observations across different levels of organization, from both a structural and functional perspective. We argue that, paradoxically, the level of cortical circuits is the least understood from a structural point of view and perhaps the best studied from a dynamical one. We further link observations about scale-freeness and fractality with evidence that the environment provides constraints that may explain the usefulness of fractal structure and scale-free dynamics in the brain. Moreover, we discuss evidence that behavior exhibits scale-free properties, likely emerging from similarly organized brain dynamics, enabling an organism to thrive in an environment that shares the same organizational principles. Finally, we review the sparse evidence for and try to speculate on the functional consequences of fractality and scale-freeness for brain computation. These properties may endow the brain with computational capabilities that transcend current models of neural computation and could hold the key to unraveling how the brain constructs percepts and generates behavior.

## Introduction

In spite of more than a century of research on the structure and function of the nervous system, we are still far from a mechanistic understanding of how biological brains solve major perceptual challenges, how they support cognition, and generate behavior (Kumar et al. 2013). We argue that current limitations stem from multiple different sources, all ultimately rooted in the “curse of complexity” (Bassett and Gazzaniga 2011). Progress beyond current limits requires the identification of fundamental principles that give rise to, govern, and take advantage of the complexity of brain networks (Skarda and Freeman 1987; Werner 2007; Friston 2010).

One major limitation emerges from the sheer size of biological brains, which are organized on multiple structural and functional levels. Since Ramón y Cajal’s seminal work on the cellular structure of neural tissue (Cajal 1995; Glickstein 2006), a considerable amount of evidence has been accumulated about nerve cells, on what is called the “microscopic level” (van den Heuvel and Yeo 2017). Neurons have been investigated in terms of their molecular and morphological structure, or their physiological and functional properties (Bullock et al. 2005). Also, much is known at the so-called “macroscopic level” (van den Heuvel and Yeo 2017; Suárez et al. 2020), spanning the global, brain-wide structural, and functional networks. Techniques such as postmortem diffusion tractography (Haber 1988; Miller et al. 2012), retrograde tracing (Markov et al. 2014; Oh et al. 2014; Zingg et al. 2014), diffusion tensor imaging (DTI; O’Donnell and Westin 2011), electroencephalography (EEG; Nunez and Srinivasan 2006), magnetoencephalography (MEG; Baillet 2017), or functional magnetic resonance imaging (fMRI; Glover 2011) have revealed a wealth of evidence for precisely organized structural connectivity and for dynamical activity patterns that correlate with perception and behavior (Uhlhaas et al. 2009).

Here, we argue that, at least when it comes to complex brains, such as that of mammals, we are currently stuck at the “mesoscopic level” of the neuronal circuits, bridging the microscopic and macroscopic levels (Mitra 2014; Khambhati et al. 2018; Huang et al. 2020; Orsolic et al. 2021). Technological constraints have arguably confined initial efforts to either the observation of individual neurons, or to the observation of global, brain-wide structural and functional patterns. Only relatively recently, with the rapid advance of technological prowess in electrophysiology and microscopy, have we witnessed an increased effort to understand the mesoscopic level of the neural circuits (Singer 1999). However, complexity, manifested at the level of neuronal circuits, is a formidable barrier to the mechanistical dissection of their function. The mesoscopic level contains a huge number of nonlinear elements interacting across vastly recurrent structures containing reentrant loops within reentrant loops (Edelman 1987). A unified understanding, linking the different scales, requires searching for fundamental structural and functional principles that govern them all. Here, we review one such fundamental principle, namely scale-freeness, which appears ubiquitous at all levels of structural and functional organization of the brain.

A second issue limiting progress in understanding brain function is the unclear relationship between structure and function. Although certain wiring patterns in brain networks may be related to wiring economy (Raj and Chen 2011; Cuntz et al. 2012; Stevens 2012; Ercsey-Ravasz et al. 2013; Wang and Clandinin 2016; Song et al. 2021) or mechanical constraints (Garcia et al. 2018; Llinares-Benadero and Borrell 2019; Wang et al. 2019), others could also entail certain functional consequences that are essential. Four decades of computational modeling suggest that structure and function are inseparable (Parr and Friston 2018; Tomasello et al. 2018; Straathof et al. 2019). The architecture of artificial neural networks vastly constrains the problems that such models can learn to solve (Nabhan and Zomaya 1994; Ciuparu et al. 2020). In the biological brain, the mammalian neocortex has a remarkably canonical structure (Thomson and Bannister 2003; Douglas and Martin 2004; Grillner et al. 2005) and a seemingly universal computation ability—the same neocortical structure processes a plethora of sensory modalities (Lakatos et al. 2007; Kayser 2010; Sperdin et al. 2010), supports cognition and decision-making (Kennerley and Walton 2011; Coutlee and Huettel 2012; Domenech and Koechlin 2015; Funahashi 2017), and enables motor control and behavior (Ebbesen and Brecht 2017; McColgan et al. 2020). The structure of the neocortical microcircuit at the mesoscale likely holds the key to its functional versatility. Thus, evidence points towards a tight relation between structure and function. It remains to be determined what the role of scale-free structure is and how it shapes useful computational processes in the brain.

A third factor hindering a mechanistic understanding of brain function is rooted in our failure to construct computational models that reproduce the principles employed by the brain (Nikolić 2009). On the one hand, we do not yet know what those principles are (Mureşan 2004; Mureşan et al. 2005a; Nikolić 2017). On the other hand, these principles have not presented themselves via any ab-initio simulation of physical or chemical processes in the nervous tissue, either. The latter is due to the sheer number and complexity of different phenomena in such tissue and our inability to discern between epiphenomena and functionally important processes. The most successful neural models today, e.g. deep learning (LeCun et al. 2015), rely on hierarchical encoding in feedforward architectures. These models could not be further away from the structural and functional principles witnessed in biological brains: Biological circuits exhibit prominent recurrence (Felleman and Van Essen 1991) and complex temporal dynamics (Singer 2021), and their way of learning does not resemble the backpropagation applied in deep learning (Singer and Lazar 2016). Efforts to study more realistic models (Gerstner and Kistler 2002; Haeusler and Maass 2007), have hit the mesoscopic bottleneck—the complexity at this level also plagues models of neural circuits. Once constructed, simulated circuits become so complex that it is difficult to make sense of their activity (Mureşan et al. 2005b; Mureşan and Savin 2007; Izhikevich and Edelman 2008; Moca et al. 2014). In the absence of additional knowledge regarding the fundamental principles that govern neural computation, it is difficult to handle dynamical complexity in realistic neural models.

In this review, we argue that one fundamental principle embedded in the very design of the brain is scale-freeness and we suggest that, once understood, this unifying principle will contribute to solving the puzzle of brain function. Systems which possess scale-freeness both in terms of their structure (fractality) and dynamics (scale-free) may be able to exhibit emergent properties that bridge spatial and temporal scales. This fundamental principle may help guide both our mechanistic understanding of brain function and the construction of more powerful computational models and algorithms, which approach the capabilities of biological brains.

To avoid ambiguity throughout this work, an overview on terminology and definitions used is given in a terminology and definitions box. In the next section, we will embed the used terminology into a broader historic and scientific context and explore how scale-invariance is expressed in natural systems. Focusing on neuroscience in Section Scale-Free Structure in the Brain at Different Levels of Organization, we will then review scale-invariance on the three levels of structural and functional organization of the brain at micro-, meso-, and macroscale. This will be linked to evidence of scale-free brain dynamics in the form of self-organized criticality (SOC), manifest from the microscale all the way to the macroscale in Section Scale-Free Dynamics. Finally, in Section Functional Role of Fractal Structure and Scale-Invariant Dynamics, the focus will be on potential implications and advantages of critical, scale-invariant dynamics, together with fractal structures. We will attempt to reconcile the collected pieces of evidence with their putative functional role, explaining why scale-freeness may be crucial for a mechanistic account of brain function.

**Table:** 

Terminology and definitions The general terminology around scale-free structure and dynamics is complex, partially overlapping, and sometimes ambiguous. Here, we give an overview of the most relevant terms as a quick reference. They have been carefully selected from a wide range of sources and attempt to unify the way people use and understand them. As a guide through these definitions, the connections and dependencies between the terms are shown in Fig. 1.		
**Scale-invariance (scale-free)** An abstract object is “scale-invariant” or (equivalently) “scale-free” if relevant features of the object remain invariant under dilations $\lambda$ of the object along a set of dimensions $x$, such as time, space, node degree, distance, etc. This results in a form of universality of the feature $f(x)$ across scales, so that $f (\lambda x)=C(\lambda )f(x)$, where C$(\lambda )$ is a universal scaling function. (Kantz and Schreiber 2003; Khaluf et al. 2017) **Power laws** A “power law” is a form of scale-invariance whereby one quantity is proportional to a power of the other, so that its scaling function reads $C(\lambda )={\lambda}^{\Delta }$ with a constant scaling exponent $\Delta$. It is notable that a power law becomes a straight line in a log–log plot with a slope equal to its exponent. (Kumamoto and Kamihigashi 2018; Crawford 2020) **Self-similarity and statistical self-similarity** An object is “self-similar” if it can be constructed as a union of rescaled copies of itself, or if each portion can be considered a scaled down image of the whole. The object is “statistically self-similar” if the rescaled parts have congruent features distribution with respect to the whole object (Sahoo and Barman 2012; Seuront 2015).	**Fractal (monofractal)** “Fractals” can be described as complex objects with fine structures at arbitrarily small scales, exhibiting some degree of approximate or statistical self-similarity, often with a recursive construction rule or definition (Strogatz 2018). **Fractal dimension** The “fractal dimension” is a scale-dependent measure of complexity that quantifies how an object fills the space. There are several flavors of fractal dimensions with different properties, but all expand the well-known notion of a 1-dimensional line or 3-dimensional cube to a wide class of sets or objects (Falconer 2014). **Multifractal** If an object obeys multiple power laws locally and all points, which are characterized by the same exponent, form a fractal, the object is called a “multifractal.” It consists of multiple intertwined fractals with individual exponents, so that the multifractal is characterized by a spectrum of scaling exponents. (Falconer 2014; Salat et al. 2017)	**Node degree, shortest path, clustering coefficient** The number of links connected to a node in a network or graph is called “node degree” (Strogatz 2001). In an unweighted network or graph, the “shortest/geodesic path” between 2 nodes is the path with the minimum number of edges, which also quantifies its length. (Watts and Strogatz 1998; Newman 2001) The “clustering coefficient” is the number of triangles in a graph divided by the number of connected triplets, where a connected triplet is a set of 3 nodes with at least 2 connected pairs. (Newman 2001) **Small-world network** A “small-world network” is characterized by short average path length that scales with the logarithm of the number of nodes and a high clustering coefficient. (Watts and Strogatz 1998) **Scale-free network** A “scale-free network” is a network with a power-law node degree distribution. In scale-free networks the scale-invariance property is expressed at the topological level in the node degree distribution. (Strogatz 2001; Barabási and Bonabeau 2003; Khaluf et al. 2017) **Fractal network** A “fractal network” exhibits self-similar branching at the structural level of the network. (Bieberich 2002)
**Scale-free (scale-invariant) dynamics** “Scale-free dynamics” refers to the activity of complex systems, which does not contain a predominant temporal scale. Hence, the same, properly scaled features can be found at any temporal scale (Khaluf et al. 2017). **Long-range temporal correlation (LRTC)** A signal is said to exhibit “LRTC” if its autocorrelation function decays at a slow pace, according to a power law, as a function of time (Meisel et al. 2017). **Order and control parameter, phase diagram** An “order parameter” is a function that distinguishes between the phases of a complex system. The arguments of an order parameter are called “control parameters.” The “phase diagram” shows the relation between the control parameters and the phase/order parameter (Sornette 2006).	**Phase transition** In a complex system, a “phase transition” is a macroscopic change of relevant observables, accompanied by a change of the order parameter, in response to an infinitesimal change in the control parameter (Sornette 2006; Bagchi 2018). In a “first order/discontinuous phase transition,” the order parameter exhibits a discontinuity. Here, the 2 states discontinuously coexist at transition boundary. The system is characterized by the presence of hysteresis and finite correlation lengths (Sornette 2006; Onuki 2007; Plenz and Niebur 2014; Rose and Dupuis 2017; Bagchi 2018). In contrast, if the order parameter is a continuous function of the control parameter, the phase transition is referred to as “second order/continuous.” The system shows scale-invariant behavior, i.e. fluctuations on all length scales, long-range correlations, and power-law scaling (Sornette 2006; Onuki 2007; Lesne et al. 2012; Plenz and Niebur 2014; Bagchi 2018).	**Critical point, criticality** The “critical point” is the coordinate in the phase diagram from where the transition between 2 states becomes continuous. A complex system at the critical point is said to be at “criticality” (Stowe 2007; Lesne et al. 2012; Watkins et al. 2016). **Self-organized criticality (SOC)** A system is said to be at “SOC” if the system is critical, and the system’s control parameters are self-driven towards the critical regime (Lesne et al. 2012; Watkins et al. 2016). **Neuronal avalanche** “Neuronal avalanches” are a special case of balanced cascades of neural activity, often with complex spatiotemporal structures. They occur naturally at criticality and are found in the superficial layers of the cortex (Beggs and Plenz 2003).

**Fig. 1:**
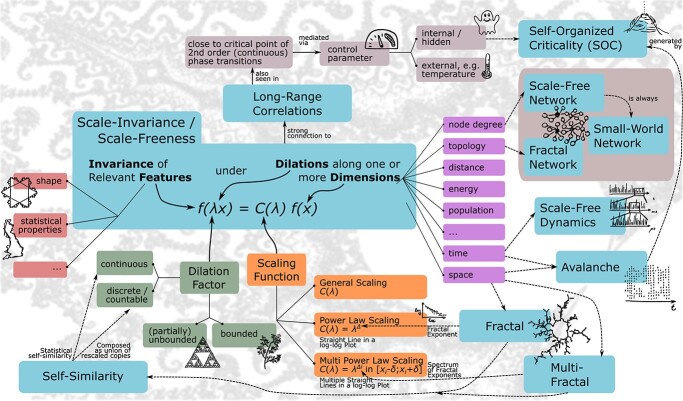
Overview on the terminology around scale-invariance, self-similarity, and fractals.

## Scale-free systems in general and in nature

Before discussing scale-free structure and dynamics in the brain, it is necessary to build a general understanding of the abstract concepts of scale-invariance, fractals, criticality, and related terms. As a quick reference, the terminology and definitions box provides an overview on these terms and highlights links and relationships between them.

To follow a consistent nomenclature throughout this article, we use the terms “scale-invariant” and “scale-free” synonymously as very broad concepts for situations where certain relevant features of an object remain unchanged when rescaling the object. There are 3 important special cases of how such a rescaling can be realized:

(i) “Geometric rescaling” changes the size of an object in the spatial domain and is connected to the definition of fractals.
(ii) “Rescaling in time” replaces a measurement interval by another one of longer or shorter duration. It is connected to the definition of scale-free dynamics.
(iii) “Topological rescaling” applied to a network changes the connectivity statistics of its nodes. For example, the scaling properties of the node degree distribution provide the basis for the definition of scale-free networks.

We now explore each of these 3 cases in more detail.

### Geometric rescaling and fractals

Scale-free structures and dynamics have been studied in vastly different fields of science for >50 years. The origins go back to a seminal publication by Mandelbrot, where he coined the term “statistical self-similarity” in observing that for certain objects “each […] portion can – in a statistical sense – be considered a reduced-scale image of the whole” (Mandelbrot 1967).

Mandelbrot compared 2 mathematical definitions of “dimension”: On the one hand, the “topological dimension” of an object is—intuitively speaking—computed by covering it with increasingly refined open sets and counting the minimally possible mutual overlaps between these sets. On the other hand, the “Hausdorff–Besicovitch dimension” measures how a geometric object behaves under rescaling. Both definitions agree for usual “well-behaved” geometrical objects and assign, e.g. a dimension of 1 to a straight line and a dimension of 2 to a square in Euclidian space.

However, taking the coastline of Britain as an example, Mandelbrot showed that the self-similarity of curves can give rise to noninteger, i.e. fractional, dimensions larger than one. Later, he generalized this to higher-dimensional cases and introduced the term “fractal” for geometrical objects whose Hausdorff–Besicovitch dimension strictly exceeds their “topological” dimension (Mandelbrot 1977; Mandelbrot and Wheeler 1983).

Defining fractality only based on the Hausdorff–Besicovitch dimension, as well as similar approaches to define a consistent “fractal dimension” that completely covers all interesting fractal phenomena, is usually considered too strict. The reason is that several mathematical objects like the boundary of the Mandelbrot set or many space-filling curves, which are typically interpreted as being fractal, cannot be identified as fractal entities using a fractal dimension because in these cases the latter is identical to the corresponding topological dimension (Shishikura 1998). Thus, intuitively the characteristic of a fractal should rather be reduced to: “A fractal is a shape made of parts similar to the whole in some way.” (Feder 1988), which often also implies invariance with respect to displacement (Mandelbrot and Wheeler 1983). More generally, the strict definition of a fractal has been replaced by the description of fractals through several or all of the following properties (Falconer 2014):

(a) Fractals have a fine structure down to arbitrarily small scales.
(b) A local or global description with traditional geometry is prevented by a fractal’s irregularity.
(c) Fractals exhibit exact, approximate, or statistical self-similarity across scales.
(d) One or more notions of “fractal dimension,” a generalization of the classical “geometric dimension,” exceeds its topological dimension.
(e) There is a simple, often recursive, construction rule.

The different notions of self-similarity under point (c) deserve some clarification: A fractal is exactly self-similar if it consists of several, properly scaled, exact copies of itself, which is possible only for abstract mathematical objects like the Koch snowflake (von Koch 1904) or the Sierpinski gasket (Sierpinski 1915). Physical objects like a real snowflake can at best be approximately self-similar due to physical constraints, which prohibit an infinite stack of smaller and smaller copies. Statistical self-similarity, on the other hand, implies that an object is composed out of smaller components, which are not its exact copies but exhibit the same statistical properties when scaled appropriately. Many real-life objects or phenomena have been found to meet these criteria reasonably well: For example, fern leaves (Campbell 1996), broccoli and cauliflower (Kim 2004), dendritic trees (Takeda et al. 1992; Smith et al. 2021), or river networks (Gupta and Waymire 1989).

### Rescaling in time and scale-free dynamics

Scale-invariance can be found across temporal scales in the dynamics of complex systems, which means that some observed signal is similar to itself on different time scales. Temporal scale-invariance is in practice—as opposed to geometric fractality—always of purely statistical nature, e.g. in phenomena based on random-walk processes (Mitra et al. 2021). The invariance is imprinted in the rules, which govern the creation of the observed signal, so that the self-similarity is only revealed in its statistical properties. For example, a particular stock chart is not self-similar, but an underlying random-walk model might be self-similar in the sense that a rescaling of both the temporal and the spatial dimension leaves the probability distribution of the change rate invariant.

### Topological rescaling and scale-free networks

In network science, a network is a graph consisting of interconnected nodes (Boccaletti et al. 2006). The degree of a node is the number of other nodes to which it is linked. The degree distribution of a network measures the probability for some arbitrarily selected node to have a certain degree. A network is called scale-free if its degree distribution obeys a power law, i.e. if the share of nodes of degree $k$ is proportional to ${k}^{-\gamma }$ for some constant parameter $\gamma$ (Barabási et al. 2001).

We must clearly distinguish between scale-free “geometry” and scale-free “networks” in the network science sense. A network may be realized physically in space, and may be geometrically self-similar, but this does not necessarily imply that it is a scale-free “network.” In the brain, e.g. we can see a geometrically self-similar structure from the micro-level up to the complex cellular networks connecting different areas (Guidolin et al. 2016), but the network (graph) itself is not scale-free.

### Scale-invariance in multiple scientific fields

Examples for the various types of scale-invariance have been observed in many different natural systems, ranging from large-scale objects in astrophysics (Press 1978; Beloborodov et al. 1998; Datta 2003; Newman 2005; Sylos Labini et al. 2009) or geology (Meng et al. 2019), over natural disasters (Newman 2005; He et al. 2016) and electric breakdown phenomena (Niemeyer et al. 1984), across plant structure (Eshel 1998; West et al. 1999a), classification and observation of species (Brown et al. 2002; Smith et al. 2003; Sizling and Storch 2004; Clauset et al. 2009), down to properties of proteins in specific organisms (Ito et al. 2000), noncoding areas of DNA (Stanley et al. 1993), and slip events in crystals (Dimiduk 2006). Even behavior of humans and animals shows scale-free properties (Hausdorff et al. 1995; Kuznetsov and Wallot 2011; Stephen and Anastas 2011; Stephen and Dixon 2011; Ravi et al. 2020) or is sensitive to fractal stimulation (Marmelat et al. 2014).

Artificial objects and phenomena exhibit scale-free properties as well, such as bodies of literature (Redner 1998; Newman 2005), subgroups of communication or power networks (Newman 2005; Holme et al. 2007), modules in logic graphs and computer chips constructed from them (Landman and Russo 1971), structure and properties of human society (Clauset et al. 2009) and organizations (Biggiero and Angelini 2015), social network dynamics (Aiello et al. 2000), or human interaction (Small and Singer 1982; Roberts and Turcotte 1998; Clauset et al. 2007). Scale-free properties have even been proven to be useful when specifically designed for technical applications (Krukiewicz et al. 2018; Minati et al. 2018). Scale-free phenomena can also be observed in the field of software programming (Hyland-Wood et al. 2005; Potanin et al. 2005). A comprehensive overview and more examples can be found in Gisiger (2001).

### Relation to power laws

There is a close connection between scale-invariance and power laws. Intuitively, correlations between events in the spatio-temporal case or localized microscopic states are expected to decay exponentially with respect to distance (Stanley et al. 1993), which—via the fluctuation dissipation theorem (Callen and Welton 1951)—would give rise to an exponential decay of observable mean values. However, for scale-invariant observables $f(x)$, a scaling relationship of the form $f(\lambda x)={\lambda}^{\Delta}f(x)$ holds, where $\Delta$ is a scaling exponent. Rescaling dimension $x\to \lambda x$ dilates the observable by a factor ${\lambda}^{\Delta}$. This behavior does not correspond to an exponential but is fulfilled by a power-law decay $f(x)\propto{x}^{-n}$, such that plotting the observable on a log–log scale reveals a straight line. A similar argumentation applies to self-similar processes (Newberry and Savage 2019). In the case of scale-free dynamics, this power law is often referred to as $1/{f}^{\alpha }$*-noise* (Press 1978). For geometric scale-invariance, the power law is essentially how the Hausdorff dimension is measured. The fact that spatial or temporal correlations do not decay exponentially, but with a slower power law, entails that these systems exhibit so-called “long-range” or “long-time correlations.”

If a single power-law exponent $n$ is not sufficient for describing the fractal system but multiple exponents $n(x)$ are required, the power law only holds locally, such that $f(x+\delta )-f(x)\propto{x}^{-n(x)}$ for sufficiently small $\delta$. These systems are called “multifractal” (Harte 2001).

Several rigorous experimental and mathematical methods exist to characterize, quantize, and critically analyze scale-free systems, either via the characteristic power-law distribution (Clauset et al. 2009; Wijnants et al. 2013; Broido and Clauset 2019; Newberry and Savage 2019) or via wavelet type approaches (Mukli et al. 2015; Nagy et al. 2017; Abry et al. 2019; Moca et al. 2021).

### Relation to (self-organized) criticality

When physical matter undergoes a phase transition from gaseous across liquid to solid state, intermolecular correlations increase: The molecules become more and more well-ordered with respect to each other. Similarly, the emergence of long-range correlations in scale-free systems raises the suspicion of being connected with a phase transition or a critical point, which marks the transition between regions in the phase diagram with distinct, separable phases, to the region of continuous co-existence of phases. At the critical point, the correlation length diverges.

In many of the examples of scale-invariance listed above, the presence of a phase transition in complex systems is difficult to identify, though. In addition, most of them are not modulated by an external control parameter, such as temperature or pressure in classical thermodynamic systems. In these cases, criticality must result from a self-organizing process. As a fundamental concept for studying such systems, Bak, Tang, and Wiesenfeld introduced SOC, where the critical point is an attractor for the dynamic evolution of the system, and studied it using their famous sandpile-model (Bak et al. 1987, 1988). Since then, SOC has become a central concept in the theory of dynamical systems, having a broad impact in various scientific disciplines over the past 30 years (Watkins et al. 2016). SOC has been observed in forest fires (Malamud et al. 1998), solar flares (Lu and Hamilton 1991), earthquakes (Bak and Tang 1989) and, most notably, in neural systems and brains (de Arcangelis et al. 2006; Hesse and Gross 2014). The connections between fractals, scale-free systems, and SOC are also well-known and established in a variety of different fields and applications, e.g. in dissipative dynamics (Hwa and Kardar 1989), proteins (Phillips 2014), scale-free and small-world networks (Massobrio et al. 2015), and many more (Bak and Creutz 1994).

Computationally it is possible to model SOC and self-similarity of many systems using cellular automata (Creutz 2012). However, among the many possible construction rules for cellular automata, scale-invariance is rare and $1/{f}^{\alpha }$ power laws come about only in a very limited but interesting set of situations (Nagler and Claussen 2005).

### Purpose and potential of scale-free systems

It is difficult to decide whether fractality, scale-invariance or SOC serve a function in all the domains in which they are observed or if they are mere epiphenomena of the respective systems (Kadanoff 1986). There are few examples where empirical data indicate a clear benefit from fractal structures. For example, the phyllotaxis of plants, which is closely related to the Fibonacci sequence, is an immediate result of an energy minimization (Shipman and Newell 2005; Okabe 2015). Maximization of organism metabolic capacity and internal efficiency via maximized exchange surface area scaling and minimized internal transport distances and times are related to fractal geometry and might be a reason for its abundance in biology (West et al. 1999b; West 2014). Also, there is a close connection between plant root fractal dimension and drought stress (Wang et al. 2009) or increased phosphor uptake in plant roots with higher fractal dimension (Nielsen et al. 1999).

Interestingly, optimization of microcircuit design has been shown to be related to Rent’s rule, linking the number of inputs and outputs to the number of internal logic gates. Here, a preference for local connections in a homogeneous circuit leads to a power-law relation: The number of inputs and outputs of a circuit is proportional to the number of internal gates. That is, the complexity of a circuit’s function is proportional to the complexity of the supporting internal structure (Christie and Stroobandt 2000). The scaling exponent is a measure of the circuit’s dimensionality (Hagen et al. 1994).

Analyzing scale-invariance, its theoretical underpinning, and its potential purpose has inspired new research approaches in many domains, and the same can be expected for its various manifestations in neuroscience. Accordingly, in the following sections we will concentrate on scale-free structures and dynamics in the brain, covering a broad range of scales and observables, together with their properties, and their relation to (self-organized) criticality.

## Scale-free structure in the brain at different levels of organization

**Fig. 2:**
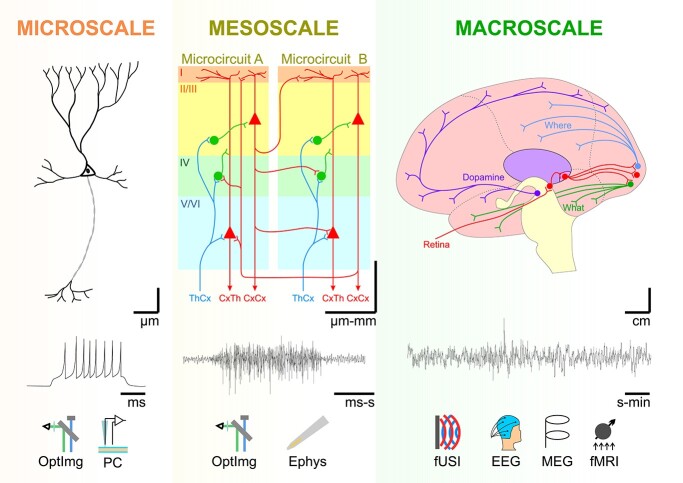
Scaling levels in the human brain. The microscale is characterized by microscopic structures, at cellular and subcellular level (μm) with dynamics expressed on very fast timescales (ms), captured with recording techniques such as optical imaging (OptImg), or patch clamp electrophysiology (PC). The mesoscale, the level of the neuronal circuits, spans a spatial range of micrometers to millimeters, with its activity being observed using optical imaging (OptImg) or extracellular electrophysiology (Ephys). Finally, the macroscale is manifest at the level of brain areas and global brain networks, spanning cm in humans and with global activity traces that encompass seconds to minutes. The latter is usually observed using functional ultrasound imaging (fUSI), EEG, MEG, or fMRI.

We will next review evidence of scale-free, fractal structure in the brain at the 3 levels of organization: macro-, meso-, and microscale, cf. Fig. 2. As will be shown, there is abundant evidence for fractality at the macro- and microscale but, due to technological limitations and its sheer complexity, we are only now beginning to reveal the mesoscale’s secrets. Building up on technological advances, recent work has shown promise to bring exciting insights into micro-connectomics that may enable fractal analysis of the connectome in the near future (Haupt et al. 2017; Zeng 2018; Friedmann et al. 2020; Scheffer et al. 2020), as without detailed knowledge of mesoscale structures and connections it is difficult to discuss about fractality at this scale.

From a structural perspective, the brain possesses scale-free properties, especially when it comes to global, inter-areal long-range connections (Katsaloulis et al. 2009), as well as to local, dendritic, and axonal arborizations (Smith et al. 2021). However, this fractal property is not the only structural feature: The geometry of brain networks also abundantly exhibits other patterns, such as small-worldness (Hilgetag and Kaiser 2004; Bassett and Bullmore 2017). Therefore, one may ask which aspects of the brain are characterized by fractality and which are not, and how these different geometric patterns determine function.

Care must be taken when interpreting results of studies related to fractal concepts (Werner 2010; Di Ieva 2016). On the one hand, fractality can be defined as the scale-free geometric property of neural structures (Falconer 2014). On the other hand, fractal dimension as a mathematical measure of fractality has also been used to characterize non-fractal data (Nikolić et al. 2008), e.g. in order to assess “complexity” of brain structures in health and disease (Cook et al. 1995; Pirici et al. 2016; Zhang and Yue 2016). Therefore, one must distinguish between usage of fractal concepts for measuring complexity and genuine fractal structure, assessed by highlighting geometric scale-invariance.

Importantly, with the exception of the microscopic level of the cells and their cellular and subcellular structures, the mesoscopic and macroscopic levels are defined here not in absolute dimensional terms, but relative to brain size. For example, the connectome of the fruit fly, which involves ~25,000 neurons and 20 million synapses (Scheffer et al. 2020), would represent the macroscale in our definition. Similarly, the whole brain connectome of the mouse (Knox et al. 2018) would also characterize the macroscale level, even if it involves 2 orders of magnitude more neurons. To more objectively delineate these scales, we consider that the macroscale involves global brain connectivity bridging the sensory and motor structures, i.e. inter-areal connectivity. The mesoscale is then defined as the intermediate level, of the local circuits, between the microscale (cellular) and macroscale levels. Furthermore, when we discuss mesoscale complexity, we usually refer to the cortex of the mammalian brain.

Traversing Fig. 2 from right to left, we will next “zoom in” on the brain, starting at the macroscale all the way down to the microscale and review and discuss evidence for fractal structure at all levels of organization. We will also discuss evidence of non-fractal structures and attempt to distinguish between the use of fractal measurements for assessing complexity versus true fractal structure.

### Macroscale

At the level of the global brain structure, there is plenty of evidence for fractality, starting with the folding of the cerebral cortex (gyrification), volumetric distribution, or the spatial distribution of the connecting fibers between brain areas. On the other hand, fractal dimension as a mathematical concept, has been abundantly used as a metric to characterize various types of macroscale structural variations in disease (Cook et al. 1995; Pirici et al. 2016; Zhang and Yue 2016), development, aging (Blanton et al. 2001; Reishofer et al. 2018), and biological sex (Farahibozorg et al. 2015). Finally, the brain-wide connectome has also been analyzed from a network science perspective (connectivity graph). Although the connectivity graph does not exhibit scale-freeness, it is interestingly related to the fractal macroscale geometry.

#### Fractal geometry at the macroscale

One of the most salient properties of the brain’s macroscale geometry is gyrification, the fractal-like folding of the cerebral cortex. It is still an open question if this geometry has some functional role, but it certainly helps to optimize the space and tissue required to connect different functional areas (Hofman 2016). Interestingly, the physical constraints during brain development alone suffice to produce such kind of structures. Indeed, the growth of gray matter embodied between more slowly growing white matter and the skull is enough to produce gyrification and folding (Herculano-Houzel et al. 2008, 2010; Mota and Herculano-Houzel 2012; Ribeiro et al. 2013; Tallinen et al. 2016).

When looking at brain geometry from an evolutionary perspective, many scaling laws have been discovered. First, a universal scaling law between gray and white matter seems to exist across species, with a scaling coefficient of ~1.23 (Zhang and Sejnowski 2000). However, during evolution, the scaling of the mass, volume, and surface area of gray matter and/or white matter (Herculano-Houzel et al. 2007, 2010; Herculano-Houzel 2012a), shows clear differences between rodents and primates (Herculano-Houzel 2012b).

In volumetric analyses, the box-counting dimensionality method was applied to T1 (Blanton et al. 2001) or multifractal analysis to T2 (Takahashi et al. 2004) magnetic resonance imaging (MRI) scans. Using the box-counting method, generally interpreted as reflecting the complexity of the cortical structure, a variety of values for the dimensionality has been reported: for the human cortex between 2.09 (Blanton et al. 2001) and 2.8 (Kiselev et al. 2003), for the cortical surface of 2.6 (Majumdar and Prasad 1988), for the cerebellum of 2.57 (Liu et al. 2003), and for the surface of white matter between 1.45 (Cook et al. 1995) and 2.3 (Free et al. 1996). The large differences in these results can be attributed to differences in methodology ([Bibr ref198][Bibr ref198]), or the type of analysis, since gray matter, e.g. can be studied in 2 (Takahashi et al. 2004; Kalmanti and Maris 2007; Farahibozorg et al. 2015; Reishofer et al. 2018; Marzi et al. 2021) or 3 dimensions (Chuang et al. 1991; Free et al. 1996; Blanton et al. 2001; Zhang et al. 2006). In the 2D case, the box-counting dimension is computed on the cortical surface, whereas in the 3D case, the entire volume is taken into consideration.

Another useful technique to map out the structure at macroscale is DTI, a special MRI tool to visualize the diffusion of water molecules through tissue. Although standard MRI scans are used to study the cortical surface (gray matter), DTI can be employed to map the structure of axonal tracts in the white matter, which are particularly conductive to the diffusion of water (Basser et al. 1994). Box-counting on these tracts embedded in the 3D space yields a dimensionality of 1.6 (Katsaloulis et al. 2009). The latter study also estimated fractal properties using lacunarity, which measures how well objects fill space. Analyzing the eigenvalues of the effective diffusion tensor, it furthermore found rotational symmetry.

#### Fractal dimension as a diagnostic metric

Although the fractal dimension of the healthy human brain has been studied to determine its structure, it has also been used as a measure of complexity in diagnosing and studying various diseases and in investigating the phenomena of development or aging. For example, the fractal dimension of the cortical surface increases in early fetal development and seems to continue till adolescence or adulthood, declining after this point with age (Takahashi et al. 2004; Kalmanti and Maris 2007; Wu et al. 2009; Farahibozorg et al. 2015; Reishofer et al. 2018). Fractal dimension of various brain structures is also smaller than average in the cases of Alzheimer’s disease (Pirici et al. 2016; Zhang and Yue 2016), amyotrophic lateral sclerosis (ALS; Zhang and Yue 2016), epilepsy (Cook et al. 1995; Lin et al. 2007a), multiple sclerosis (Lin et al. 2007a; Pirici et al. 2016; Zhang and Yue 2016), atrophy (Pirici et al. 2016; Zhang and Yue 2016; Lin et al. 2007b), stroke (Pirici et al. 2016; Zhang and Yue 2016), and autism and schizophrenia (Bullmore et al. 1994; Zhao et al. 2018). It is important to note that in all these cases, a lower fractal dimension is correlated with loss of brain function in one form or another, hinting at its importance. The one exception to this rule seems to be the bipolar disorder, which is associated with a slight increase of the fractal dimension compared with a healthy control group (Bullmore et al. 1994).

#### Global brain connectomics

From a geometric perspective, complex networks on the macro-scale show high resemblance to micro-scale neuronal connections (Guidolin et al. 2016). However, when talking about global brain connectomics, from a network science perspective, one usually refers to the inter-areal network of the brain—the connection graph between functional areas, or other regions of interest. As discussed by Bassett and Bullmore (2017) the brain has been considered a small-world network (graph) in the last 2 decades (Hilgetag and Kaiser 2004; Sporns and Zwi 2004; Bassett et al. 2006; Bassett and Bullmore 2006; Vaessen et al. 2010; Sporns and Betzel 2016).

Small-worldness means large clustering and short path lengths in the network. Such network characteristics can be constructed, e.g. through a hierarchical modular structure, which is clearly present in the brain (Hilgetag et al. 2000; Hilgetag and Kaiser 2004; Meunier et al. 2010; Bullmore and Sporns 2012; van den Heuvel et al. 2012; Sporns and Betzel 2016).

New tract tracing studies have shown that these networks are much denser than originally considered (Markov et al. 2013, 2014; Oh et al. 2014; Song et al. 2014; Rubinov et al. 2015). The inter-areal cortico-cortical structural network contains ~66% of all connections that can exist for the macaque (Markov et al. 2013, 2014), and ~97% for the mouse (Gămănuţ et al. 2018), hence talking about small-world structures in these dense binary networks is not really meaningful. It is important to study brain networks as weighted graphs, especially because the weights can vary over 6 orders of magnitude (Ercsey-Ravasz et al. 2013; Markov et al. 2014) and weak connections are also important (Horvát et al. 2016; Gămănuţ et al. 2018). Bassett and Bullmore redefined the small-world measure for weighted graphs and developed a version that allows the comparison of graphs with different densities (Bassett and Bullmore 2017). The final conclusion was that, when looking at these weighted graphs in the mouse and macaque, obtained via tract tracing by Markov and others, the weighted measures still indicate the presence of small-worldness (Markov et al. 2014; Oh et al. 2014; Rubinov et al. 2015; Gămănuţ et al. 2018).

#### Relation between macroscale geometry and global brain connectomics

In the brain, the realization rule for the locally modular, hierarchical structure that also provides small-worldness and its characteristics is the exponential distance rule (EDR), which shows that the number of inter-areal axons decreases exponentially with their length (Ercsey-Ravasz et al. 2013). This is directly related to minimization of wiring cost, but most importantly it assures that areas close to each other have strong connections, and areas that are far from each other have much weaker connections. The EDR most probably is a result of a physical process of axon growth, because it is valid both on micro-scale in the gray matter and macro-scale in the white matter. It is interesting that looking at the decay rate parameter in different species, it almost perfectly coincides in the case of the gray matter, where its value is $l=4.61\ {\textrm{mm}}^{-1}$ in mouse, $l=4.46\ {\textrm{mm}}^{-1}$ in macaque (Horvát et al. 2016). For the white matter, the $l$-values become almost independent of the species if distances are normalized with the average distance between areas (Horvát et al. 2016).

We can already see the connection between the macroscale geometry and connectomics of the brain. Although the axon growth during development assures the tension in the white matter that leads to cortical folding and the fractal structures, the EDR provides a locally modular, hierarchical small world structure, and many other interesting properties of the inter-areal network.

#### Connectivity principles and the link between structural and functional connectivity

Synaptic connectivity in the brain seems to obey some interesting connectivity principles, some being expressed on both the macro and the mesoscale. For example, neural wiring incorporates fractal principles (Hofman 2016; Schröter et al. 2017) because the brain is dominated by a tradeoff between wiring economy, on one hand, and efficient communication between neurons, on the other (Bullmore and Sporns 2012; Song et al. 2021). This results in a modular small-world organization (Watts and Strogatz 1998; Bassett and Bullmore 2006, 2017) beneficial enough to have been preserved across evolution from simple organisms, such as *Caenorhabditis elegans* and *drosophila*, to humans (Achard 2006; Gu et al. 2015; Schröter et al. 2017).

Sparse connectivity, long-tailed synaptic weight distributions, small-world network topology, rich clubs, and hub neurons seem to have positive computational consequences but are expensive to maintain. For instance, in *drosophila* and *C. elegans*, hub neurons and rich clubs have been mapped to different behaviors and to the organism’s ability to switch between them (Kawano et al. 2011; Zhen and Samuel 2015).

**Fig. 3:**
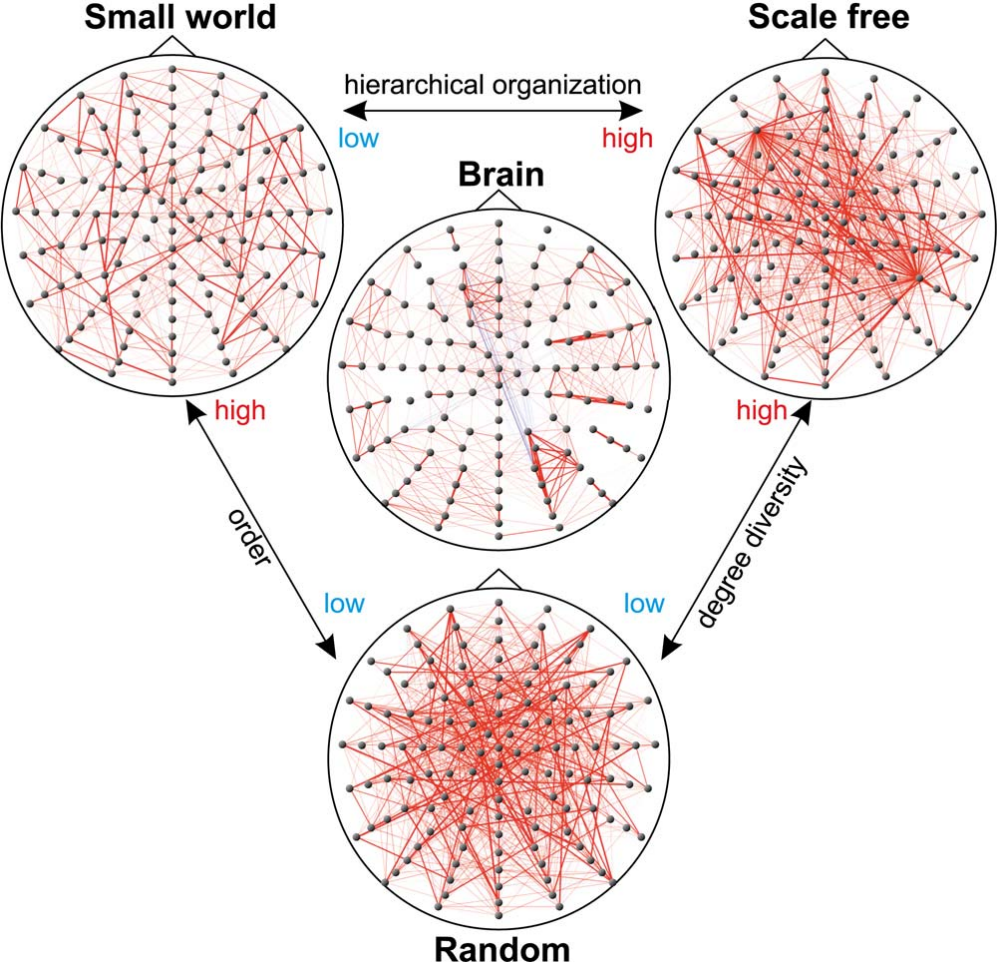
Organization of the brain network. The brain network topology is in between different network types.

Regardless of the observations presented above, the brain does not strictly obey a scale-free property in the formal way of topology: The connectome of the brain seems much richer in diversity, enhanced by the synaptic weights, resembling a combination of characteristics of different models such as random networks or locally connected networks, and exhibits high clustering with hubs similar to scale-free networks (Stam 2014; Khaluf et al. 2017). This strongly indicates the presence of hierarchical modularity, cf. Fig. 3, which was inspired by Stam (2014) and is based on EEG data (Dolean et al. 2018). The low-average node distance and hubs are estimated to play a critical role in the emergence of cognition and cognitive control functions as the hubs, classified as control nodes, drive large populations of local neurons towards a variety of states, and enrich the phase space of reachable attractors. This enables the system to perform both modular and specialized information processing, locally relative to neighborhoods, or globally distributed (Achard 2006; Gu et al. 2015), enabling specialized regions to share computational resources and information for complex problem solving.

#### Rent’s rule

An investigation on the connectivity of *C. elegans* reveals that the organism obeys a surprising type of scale-free and fractal organization principle, namely Rent’s rule of connectivity (Bassett et al. 2010). The rule states that the number of neurons within a group and the number of connections to that group are bound by a scale-free fractal relation. Rent’s rule has been first observed and studied in the field of large-scale integrated circuit design where it predicts the number of required pins for a group of logic gates to communicate with the rest of the circuit (Christie and Stroobandt 2000). The fact that it applies to *C. elegans* is surprising. Even more surprising is that Rent’s rule applies also to the connections between the modules of the fly brain where the exponent is typical to a system optimized for Packing (Scheffer et al. 2020). In general, Rent’s rule seems to occur naturally in any system that seeks to optimize spatial packing or wiring lengths (Scheffer et al. 2020). It remains a challenge to determine if Rent’s rule also applies to more complex structures, such as the mammalian neocortex and if it is expressed on other levels of organization, such as the mesoscale.

### Mesoscale

Among the 3 spatial scales, at least for the case of the mammalian cortex, the mesoscale is the most difficult to investigate due to the intricate cell structures that fill the cortical space. Space constraints stemming, from wiring length minimization (Bullmore and Sporns 2012; Schröter et al. 2017), render the cortical space notoriously packed (Helmstaedter 2013; Motta et al. 2019). A recent high-density 3D neuropil reconstruction of layer IV of the mouse somatosensory cortex from electron microscopy (EM) stacks have shown that from the total traced path length ~66.6% are non-proximal axons, 20.5% spine necks, 10.3% dendrites, and only 2.6% are cell bodies (Motta et al. 2019). Local connectivity is estimated to be sparse, regardless of the sheer complexity of neuronal connections and the densely occupied cortical space (Schröter et al. 2017). Coupled with our limited capability of acquiring connectivity data on large enough volumes (Mitra 2014; Schröter et al. 2017; Motta et al. 2019; Cano-Astorga et al. 2021) this explains why the mesoscale connectivity is so difficult to probe in the mammalian cortex.

Not only is solving the brain circuits’ synaptic connectivity extremely challenging (Lichtman and Denk 2011; Helmstaedter 2013), but the analysis framework to integrate (Mitra 2014) and deal quantitatively with such data is still at its very beginnings (Schröter et al. 2017). In a 2009 manifesto, several authors emphasize our “sparse” knowledge of cortical circuitry and propose a coordinated effort with an ambitious goal: to determine the neuroanatomical connectivity in model organisms specifically at mesoscopic scale (Bohland et al. 2009). For these reasons, with few exceptions highlighted below, the fractal and scale-free cortical aspects of the mesoscale have yet to be addressed. Even though the fractal and scale-free circuit properties are little understood, there is a relatively large body of literature on the general mesoscale principles governing the organization and wiring of the nervous systems in general and of the neocortex in particular. We briefly discuss these principles in the following.

#### Structural principles and local circuit organization

Among the principles that are observable at the interface between meso- and macroscale, the six layered laminar organization is perhaps the best known. It is mostly prominent in the mammalian neocortex (Harris et al. 2019) but is, to a lesser extent, also present in the reptile cortex (Mountcastle 1997) and, as a recent study shows, even birds (Stacho et al. 2020). At the same spatial scale, another principle of organization seems to be the area-dependent cell body density. For instance, in monkeys a particularly high neuronal density is found in visual areas V1 (Collins et al. 2010). Importantly, it has been shown in the macaque cortex that layer III pyramidal soma density is strongly correlated with cortical connectivity features that support function (Beul and Hilgetag 2019).

Another interesting principle of organization at mesoscale is the apparent canonicity of neocortical microcircuits in mammals (Thomson and Bannister 2003; Douglas and Martin 2004; Grillner et al. 2005). The same neocortical microcircuit seems to be repeated all over cortex, covering different sensory modalities and motor or integrative areas. It has been therefore speculated that the neocortical microcircuit is a canonical building block, with seemingly universal computation capabilities (Haeusler and Maass 2007; Markram et al. 2015). Interestingly, although the dendritic and axonal arbors of a local microcircuit may remain fixed, dynamic plasticity can occur to reconfigure the exact synaptic connections within such a structure (Kalisman et al. 2005). It remains a challenge to determine if and how fractal organization principles are manifest at the level of individual microcircuits and, even more so, to figure out how microcircuits coalesce into groups, and groups of groups of microcircuits, aggregating into brain areas. Perhaps a key to understanding brain function is precisely to reveal the scaling principles within and across microcircuits.

#### Mapping connectivity in local circuits

In terms of avenues for probing local cortical connectivity, there are at least three possible approaches. First, neural activity can be monitored to investigate specific connectivity patterns among groups with a restricted number of neurons. Using 2-photon calcium imaging and retrograde tracing, the dynamical changes in connectivity were measured down to the level of synaptic boutons, during behavior (Kim et al. 2018). The same technique can be used to reveal specific connectivity patterns. Electrophysiology studies revealed that layers 2/3 pyramidal neurons connect preferentially to neurons that receive common synaptic input (Morgenstern et al. 2016) and that neurons with similar responses connect preferentially (Cossell et al. 2015).

Second, EM studies focus on the dense anatomical reconstruction of cortical volume. The method allows for an extremely detailed mapping down to the level of synaptic boutons as well. Due to this high-resolution, it is also very demanding in terms of computing power and manual work when applied to macroscopic volumes. Motta et al. managed to reconstruct a 500,000 μm^3^ volume from the L4 barrel cortex to such detail that they were able to infer specific connectivity within the circuit (Motta et al. 2019). For example, inhibitory axons preferentially innervate certain cell compartments. They even found traces of synaptic plasticity in the structure of the synaptic boutons. In much smaller volumes of ~500 μm^3^, another recent EM study investigated the synaptic connections in human BA21 layers II–III cortical tissue (Cano-Astorga et al. 2021), showing that 93% of the synapses are asymmetric (mostly excitatory) as opposed to only 7% symmetric (inhibitory), in agreement with the literature estimates of 90–95% of asymmetric synapses. Recently, the MICrONS consortium of laboratories has managed the most extensive EM mapping to date in a volume of about 1 mm^3^ (MICrONS Consortium et al. 2021). As with other EM studies, the ability to deal with large volume is limited by the 3D reconstruction methods used to analyze the data (Turner et al. 2020; Cano-Astorga et al. 2021).

Third, following Bohland et al. (2009), several mesoscale mapping projects have been initiated. Some of these projects actually cover both the mesoscale and the more global organization of local mesoscale circuits into full brain connectomics (macroscale). Three of the most important initiatives rely on laser microscopy and tracing (Osten and Margrie 2013): The Allen Mouse Brain Connectivity Atlas (http://connectivity.brain-map.org; Oh et al. 2014; Harris et al. 2019), the Brain Architecture Project (http://brainarchitecture.org), and the Mouse Connectome Project (https://cic.ini.usc.edu; Zingg et al. 2014). For the mouse brain, these three initiatives register the coronal section images using the Allen Mouse Brain Atlas, which makes the diverse battery of tracers used by each project comparable (Osten and Margrie 2013). This greatly enhances the interpretability of the results. As with the dense reconstruction in EM, these approaches rely on technical advances to reconstruct volumes from stacks of images.

In another attempt for tracing mesoscale, neurons and synapses are labeled with genetic barcodes followed by sequencing the segmented tissue (Kebschull and Zador 2018). With barcoding, large numbers of individual neuron projections can be mapped at cellular resolution with high throughput (Chen et al. 2019). The resolution limiting factor is the ability of barcoding individual cells and the volume of the tissue to be sequenced. Compared with microscopy, barcoding provides different information. Although it loses the ability to recover the exact morphology, it provides information on the molecular expression profile of the neurons. In addition, it can properly resolve not only local but also long-range connections, the latter being problematic in microscopy as distance increases (Zador et al. 2012).

#### *Functional organization* versus *anatomical connectivity*

A wide body of literature has been dedicated to the functional organization at the mesoscale, whereby in multiple cortical systems neuronal circuits seem to be spatially organized according to some functional rule. Here, we will review the concepts of minicolums, columns, and hypercolumns, based on the columnar organization principle introduced by Mountcastle (Mountcastle 1997). However, we will also discuss more recent evidence, which challenges a direct relation between functional and anatomical organization.

A modular organization of the nervous system was proposed in many species. Cortical minicolumns (Mountcastle 1997) and the barrel cortex (Petersen 2007; Feldmeyer et al. 2013) are good examples. Even birds seem to have columnar-like structures in the Wulst nuclei and in the dorsal ventricular ridge (Stacho et al. 2020). In general, modules are defined either as groups of neurons with similar response properties or groups of cells that have a repetitive structure (Buxhoeveden and Casanova 2002).

Cortical minicolumns are defined as structures with both horizontal laminar organization and radial vertical organization of ontogenetic origin (Buxhoeveden and Casanova 2002; Rakic 2007), which have been observed as early as 1938 (Lorente de Nó 1938). Evidence for their existence was later found and systematized by Mountcastle (1957). Minicolumns were considered to be the basic anatomical unit of the cortex (Buxhoeveden and Casanova 2002). They consist of a stereotypical vertical structure with 80–100 neurons, but there a lot of heterogeneity in size and composition has been reported across areas and species (Mountcastle 1997; Buxhoeveden and Casanova 2002; Rakic 2008). On a larger spatial scale, functional columns were proposed to span ~300–600 μm in diameter (Favorov and Diamond 1990; Mountcastle 1997), being composed of ~80 functionally similar minicolumns connected by short-range horizontal connections (Mountcastle 1997). For instance, in the visual system, orientation preference and ocular dominance columns have been studied extensively. Columns are prominent also in the association areas, in the somatosensory, auditory, and motor cortices. Finally, in the visual system, the functional hypercolumn was proposed to span ~1 mm in diameter, bundling a set of columns that cover all possible values of a modality in a region of the receptive field and thus its response completely characterizes that region with respect to the modality (Hubel and Wiesel 1977; Mountcastle 1997). Interestingly, the columnar organization has a nested self-similar, fractal-like, functional structure where minicolumns are bundled in columns that form hypercolumns, which in turn are repeated across the cortical surface in a certain area. It has been argued that this “intermittently recursive mapping” organization allows for several variables to be mapped together onto the 2D cortical surface (Mountcastle 1997).

Although functional organization clearly shows columnar organization in many brain systems, the relationship between functional columns and anatomical connectivity is little understood. More recent evidence indicates that cortical columns are not physical entities, and that clusters of functionally related neurons do not necessarily align with underlying anatomical structures (Amorim Da Costa and Martin 2010). In addition, it must be emphasized that although synapse type can be inferred in detail using EM anatomical investigations (Motta et al. 2019; Cano-Astorga et al. 2021), hubs and rich clubs are identified at mesoscale based on their activity probed, for instance, with patch clamp experiments (Perin et al. 2011). No EM study has managed to identify hub neurons solely on the basis of their anatomical connectivity (Schröter et al. 2017). At the same time the functional consequence of topological connectivity is difficult to test (Mitra 2014). Thus, the relation between the anatomical connection/structure and function is not yet fully understood at the mesoscale (Mitra 2014; Kim et al. 2018).

### Microscale

At the level of single neuronal cells there is already an obvious fractal structure in the shape of their axons and dendrites. Consequently, this has been the focus of those studies, which have examined microscale fractality (Tosevski et al. 2008; Smith et al. 2021). In particular, the fractal dimensionality of dendritic arbors and its functional importance are topics of contentious debate. On the other hand, the fractal structure and distribution of neuron-specific subcellular components (synapses, ion channels, and receptors) remains underexplored.

Among the first attempts to characterize the fractal structure of neurons were the studies by Smith et al. using fractal analysis on 2D contours of pyramidal cells (Smith et al. 1989, 1996). Focusing again on morphology, Caserta et al. explored the fractal dimensionality of retinal ganglion cells to provide a better classification of retinal cell subtypes (Caserta et al. 1995). Similarly, Tosevski et al. used fractal analysis on a series of 2D images of Golgi-stained pyramidal neurons from human basolateral amygdalae (Tosevski et al. 2008), finding significant differences between the fractal dimensionality of dendritic arborizations of 2 distinct pyramidal cell subtypes. Fractal dimensionality was also evaluated for stalked and islet neurons’ dendritic arbors (dorsal horn and substantia gelatinosa) across mammalian species (rats, cats, monkeys, and humans), arguing that these variations may serve as a useful explanation of the differences in somesthetic sensibility across these species (Milošević et al. 2007). Most of these studies however used fractal dimensionality as a metric to characterize structure, without ascribing any functional role to it.

The work of Takeda et al. marks an early attempt to link fractal structure of neuronal dendrites and neuronal growth (Takeda et al. 1992). They studied Golgi-stained Purkinje cells from rats aged 5–50 days and found that fractal dimensionality $D$ of these cells increases sigmoidally. Similarly, Bernard et al. used fractal analysis on oligodendrocytes, showing that fractal dimensionality is a reliable estimator of the stage of their differentiation (Bernard et al. 2001). Alves et al. used fractal dimensionality as a parameter to find a model that more accurately describes neurite outgrowth in the dendrites of Purkinje cells and cortical axon terminals (Alves et al. 1996).

Following a different direction, Karperien et al. published a comparative review of different fractal dimensionality and lacunarity analysis techniques applied to microglia (Karperien et al. 2011). The authors show that fractal dimensionality and lacunarity change with the different degrees of microglial activation: The cells themselves change their morphology when they activate.

Connectivity modeling approaches were typically at the forefront during the 2000s and 2010s. Cuntz et al. and Wen et al. investigated the degree to which fractal dimensionality is related to dendritic shape and connectivity with regards to optimal wiring and maintenance costs (Wen et al. 2009; Cuntz et al. 2012). More recently, Smith et al. produced 3D images of Golgi-stained dorsal CA1 pyramidal cells using confocal microscopy (Smith et al. 2021). Fractal analysis was then used to characterize the dimensionality of the dendritic arbors. They argued that neurons with higher fractal dimensionality are more expensive for the organism to build and maintain. Previously, Cuntz et al. already hinted at this (Cuntz et al. 2012). This means that the brain must make a tradeoff between connectivity and energy expenditure.

From a medical perspective, there is evidence for altered dendritic structure in pathology, e.g. in rodent models with mutated autism spectrum disorder (ASD) related genes (Martínez-Cerdeño et al. 2016). The morphology of astrocytes was characterized using fractal analysis in patients with several afflictions, such as Alzheimer’s disease (AD) and stroke (Pirici et al. 2009, 2011). It was shown that it is possible to reliably differentiate between astroglial subtypes and that there is a significant difference between the fractal dimensionality of astroglia in AD patients when compared with patients who have suffered an ischemic or hemorrhagic stroke.

Taken together, these studies suggest strong functional and developmental correlations for fractality at the microscale level. It remains to be seen whether the nervous system actively relies on microscale fractality for computations. A challenge for future studies, involving experimental techniques and detailed compartmental modeling, will be to determine how fractal arborizations of dendritic and axonal trees contribute to neural computations.

## Scale-free dynamics

Next, we will focus on the manifestation of scale-invariance in brain dynamics. As opposed to the structural analysis, here we will take the reverse journey, zooming out from the microscale and up to the macroscale. As we discussed concepts such as fractal geometry and network properties in the context of brain structure, here we will review evidence of scale-invariance in the dynamical patterns generated by such structures. More specifically, we will relate the concepts of scale-free dynamics with SOC.

First, we will review evidence of scale-free dynamics at the level of cellular structures, more precisely in ion channels, and investigate the dynamics of membrane potential fluctuations in single neurons. Then, we will focus on SOC at the mesoscale, as it was shown to occur in the local-field potential (LFP) recorded in the extracellular space. Finally, we will scrutinize how these granular levels of organization reflect on the global brain state, as measured by EEG and MEG at the level of brain areas. Finally, we will review evidence that the environment also involves certain scale-free properties that impact behavior, imposing constraints on the array of dynamical control patterns the brain must generate to successfully steer an organism in its environment.

### Ion-channel kinetics

In the late 1980s and early 1990s there has been a heated debate about which model most accurately describes ion-channel kinetics. By measuring the times between state changes, commonly referred to as “dwell times” (Sansom et al. 1989; Qin and Li 2004) various families of models were employed, based on diffusion, Markov properties (“Markov Model”), and fractality (“Fractal Model”). In a Markov Model, the ion channel is modeled as a Markov chain, and the states of the ion channel are modeled as states in the Markov chain (Sigg 2014). The easiest possible Markov Model consists of only 2 states, one representing an open channel and the other representing a closed channel. Transitions between the states happen with fixed transition probabilities (Tveito et al. 2016). Typical Markov Models of ion channels incorporate more states representing an open channel than a closed one (Siekmann et al. 2011) or have additional “inactive” states (Tveito et al. 2016). Such models typically describe the behavior of the ion channel with more accuracy (Siekmann et al. 2012). However, a larger number of states do not always guarantee a better model (Korn and Horn 1988). Markov Models in which the closed state of the ion channel is modeled using from 2 up to 11 closed states, best fit experimental data (Sansom et al. 1989). However, several studies (Liebovitch et al. 1987; French and Stockbridge 1988; Millhauser et al. 1988; Roncaglia et al. 1994; Fuliński et al. 1998; Cavalcanti and Fontanazzi 1999; Kochetkov et al. 1999; Lowen et al. 1999; Mercik et al. 1999; Varanda et al. 2000; Kazachenko et al. 2007; Chui and Fyles 2010; De-Jiang et al. 2010; Silva et al. 2021) take issue with the memoryless-ness inherent to the Markov model, arguing in favor of a model that also incorporates long-term memory.

This led to the development of the first fractal model (Liebovitch et al. 1987), which has 2 states: one representing the case that the ion channel is closed, the other representing the case that it is open. The kinetic rates, defined as the time that it takes for an open channel to close and vice versa, are then modeled as a function exhibiting power-law behavior (Liebovitch et al. 1987). Even though the authors openly admit that their fractal model is not always the best model to explain the kinetics of ion channel opening and closing, this work started an intense debate about whether fractal or Markov models should be preferred (Korn and Horn 1988; McManus et al. 1988, 1989; Liebovitch 1989, 1994; Sansom et al. 1989; Varanda et al. 2000; Liebovitch et al. 2001). This debate did not yield a clear conclusion in favor of either of the 2 frameworks in the sense that certain aspects of ion-channel kinetics, like the long-term memory effects, are better explained by fractal models (Roncaglia et al. 1994; Cavalcanti and Fontanazzi 1999; Varanda et al. 2000), whereas other aspects, such as open and closed time distributions, are better explained by Markov models (McManus et al. 1988; Sansom et al. 1989). Regarding these results, it might seem peculiar why this debate arose at all if the 2 models simply explain different aspects of ion-channel kinetics and none of the 2 models can be favored in general. In 2001, Liebovitch gave a summary, describing how the debate came to life and what the main issues were: “Since each method has a different goal, some arguments talk past, rather than address each other. The goal of the nonfractal method is to determine the parameters of the kinetic diagram that best match the data. The goal of the fractal method is to determine the physical properties of the ion channel protein. The fractal approach has questioned whether the assumptions used in the nonfractal analysis are a valid representation of the physical properties of ion channel proteins.” (Liebovitch et al. 2001).

Today, both fractal models (Fuliński et al. 1998; Kochetkov et al. 1999; Mercik et al. 1999; Kazachenko et al. 2007; Chui and Fyles 2010; Moezzi et al. 2016; Borys 2020; Wawrzkiewicz-Jałowiecka et al. 2020a, 2020b) and Markov or randomized models (Wawrzkiewicz et al. 2012; Sigg 2014; Siekmann et al. 2016; Silva et al. 2021; Zifarelli et al. 2021) are accepted and used for the investigation and modeling of ion channels and their kinetics. Although many of the aforementioned models have been tested on simulated data (Moezzi et al. 2016) and cultured neuronal cells (Liebovitch et al. 1987; Sansom et al. 1989; Varanda et al. 2000), we are not aware of publications that attempt to validate these models on in vivo electrophysiological data.

### Membrane potential fluctuations and spiking events

Scale-free dynamics has been observed at the level of single neurons, in the fluctuation of the membrane potential between spiking events (Seseña-Rubfiaro et al. 2014; Johnson et al. 2019), in the inter-spike-time interval (ISI) statistics (Musha et al. 1983), and in the relationship between membrane fluctuations and spiking events (Rubfiaro et al. 2017). Methodologically, scale-free dynamics in various time series has been frequently probed by using detrended fluctuation analysis (DFA; Peng et al. 1992). The method computes a fluctuation function, which becomes a power law for signals exhibiting LRTC (refer to the terminology panel). Other techniques used to characterize scale-free dynamics include multiscale entropy (Costa et al. 2002), and methods based on avalanche characterization (Johnson et al. 2019) or rate estimate convergence (Bhattacharya et al. 2005).

#### Membrane potential fluctuation

Several studies have focused on examining membrane fluctuations recorded either from the peripheral nervous system, or in simpler animal models. As early as 1965, Verveen and Derksen have shown that fluctuations of the membrane potential in the frog axon exhibit $1/f$ scaling of the power spectrum (Verveen and Derksen 1965). Using both DFA and multiscale entropy analysis, Li et al. demonstrated that the renal sympathetic nerve activity in rats displays LRTC, which are expressed under wakefulness but are impaired by anesthesia (Li et al. 2008). In a 2014 study, the inter-spike membrane potential fluctuations were also analyzed using DFA in the pacemaker F1 neuron of the garden snail, “Helix aspersa” (Seseña-Rubfiaro et al. 2014). From the DFA analysis, they then determined the scaling exponent and found a significant relation between the latter and the ISIs of the corresponding data segments (Seseña-Rubfiaro et al. 2017).

A recent study investigated subthreshold membrane potential fluctuations in the visual cortex using whole-cell current-clamp recordings of pyramidal neurons of turtles (Johnson et al. 2019). By adapting the framework for neural avalanche analysis, scale-free properties of the subthreshold membrane fluctuations were found, consistent with critical branching. Importantly, membrane fluctuations were not matched by the LFP statistics, demonstrating that micro- and mesoscale fluctuations may occur independently.

#### Spiking event statistics

When analyzing the statistics of spiking events, Lewis et al. have shown that Fano factors of extracellular spike counts of medullary sympathetic neurons obey a power-law distribution as a function of window size, indicating LRTCs (Lewis et al. 2001). Both LRTCs and the power-law distribution are disrupted by random shuffling of inter ISIs. This demonstrates genuine statistical self-similarity in the ISI structure. Similarly, a $1/f$ scaling has been observed in the power spectrum of the ISI modulation in a completely isolated tonically autoactive neuron of the African giant snail (Musha et al. 1983). Blesić et al. have further shown that in the firing of the dorsal horn neurons, the signal obtained by sampling successive ISI durations displays a multifractal nature, with 2 different power-law scaling behaviors (Blesić et al. 2005). A similar result was obtained in human hippocampal-amygdala complex neurons (Bhattacharya et al. 2005). Guo et al. have found that long-term correlations characterize the ISI sequences of multiple classes of mouse hippocampal neurons (Guo et al. 2007). These scale-free properties change with behavioral state, such as slow-wave sleep and active exploration, with the latter showing stronger LRTCs.

#### Relation between observations in single neurons and in their embedding circuits

Accumulating evidence suggests that LRTCs occur in the activity of single neurons in various animal species and neural systems. Importantly, such properties have been observed both in cells embedded in neural circuits, including cortex, and in spontaneous firing of isolated units, receiving no input. Therefore, it is yet unclear what mechanisms underlie scale-free dynamics in single units. On one hand, this could be the result of larger population dynamics in neural circuits generating neural assemblies (Bhattacharya et al. 2005). On the other hand, recent evidence suggests the relationship between the single neuron’s and local circuit’s dynamics is not trivial (Johnson et al. 2019). Furthermore, scale-free dynamics has been observed even in isolated neurons (Musha et al. 1983), and may be the result of excitability fluctuations or complex interplay of ion channel dynamics (Seseña-Rubfiaro et al. 2017). Future studies should elucidate if LRTCs at the microscale originate from cellular or network sources, or both, and if these distinct sources have different characteristics and entail distinct functional consequences.

### SOC at the mesoscale

The concept of SOC in neuroscience is inspired from statistical mechanics and is meant to capture the dynamics of complex systems with continuous phase transitions, for which the critical point region is an attractor. For these systems, the corresponding control parameters are self-driven by internal control parameters towards the absorbing state that maintains itself once entered (Bak et al. 1988; Bak and Creutz 1994). The SOC hypothesis is reviewed in detail by Plenz (2014), Plenz and Niebur (2014), and Wilting and Priesemann (2019). Inspired by the experimental work of (Beggs and Plenz 2003) numerous experimental and theoretical studies have begun to address the hypothesis of SOC in brain dynamics. This is of particular interest, as SOC is believed to have important implications for information processing (Haldeman and Beggs 2005; Kinouchi and Copelli 2006; Beggs 2008; Tanaka et al. 2009; Shew and Plenz 2013).

Many experimental studies on SOC at the mesoscale rely on negative local-field potential (nLFP) events. nLFPs represent bursts of synchronized activity, which were initially observed in mature organotypic cultures and acute slices of rat cortex (Aertsen et al. 1996; Plenz and Kitai 1996; Karpiak and Plenz 2002). Cascades of successive time bins, in which at least one nLFP exceeds a certain threshold, were observed experimentally. Avalanches are a special case of balanced cascades, with a complex spatiotemporal configuration, which occur naturally at criticality when activity propagates in a stable manner (Beggs and Plenz 2003; Plenz 2014). Oscillations and neuronal avalanches may be a natural consequence of synchrony required for neurons to communicate (Plenz 2014). As a target neuron needs to integrate the firing of several afferent neurons in a short time window in order to fire, it is essential that the system does not explode. At the same time, the system is also required to respond to input perturbations, such that a tradeoff is born between stability and excitability (Mureşan and Savin 2007). Arguably, systems at or close to criticality fulfill this tradeoff (Bertschinger and Natschläger 2004; Williams-García et al. 2014).

Avalanches were first discovered in rat organotypic cultures (Beggs and Plenz 2003), where they occur spontaneously (Beggs and Plenz 2003; Gireesh and Plenz 2008; Plenz 2014), and then in vivo in awake monkeys (Petermann et al. 2009; Yu et al. 2011), anesthetized cats (Yu et al. 2011), mice (Ma et al. 2020), and even in resting-state human MEG (Shriki et al. 2013). They are reported to encompass mostly the superficial layers 2/3 of cortex (Beggs and Plenz 2003; Stewart and Plenz 2006; Petermann et al. 2009; Plenz 2014; Ma et al. 2020; Plenz et al. 2021), where the balanced excitation and inhibition and the lateral connectivity are instrumental in their generation and maintenance (Plenz 2014; Plenz et al. 2021).

#### Relation between avalanches and oscillations

The relation between the intricate landscape of nested oscillations and avalanches has been extensively reviewed in Plenz (2014). Both are reflections of cortical synchronization and both pertain to cortical dynamics and information processing (Buzsaki 2006; Plenz 2014). Although the 2 phenomena must coexist, the relation between them is not yet fully understood. In cultures, it has been shown that avalanches organize in nested oscillations during development of layers 2/3 (Gireesh and Plenz 2008) and that they maximize the diversity of broadband phase synchronization below 50 Hz (Yang et al. 2012). Consequently, one opinion is that avalanches are engulfed in oscillations whose transient phase locking results from their scale-free organization (Plenz 2014). Conceptually, the separation of timescales could explain how slow, wide-spread, and high amplitude oscillations that seemingly constrain the dynamics of the system can coexist with avalanches. The argument is that the relaxation time inherent to avalanches is much shorter than the slow oscillations, therefore the two do not interfere (Plenz 2014). This has been shown to be the case experimentally (Plenz 2012) and in simulations (Poil et al. 2012). Moreover, the same simulations indicate avalanches as the reason for the long-range critical scaling laws of oscillation amplitudes found in humans (Poil et al. 2012; Palva et al. 2013).

#### Scale-free properties of avalanches

**Fig. 4:**
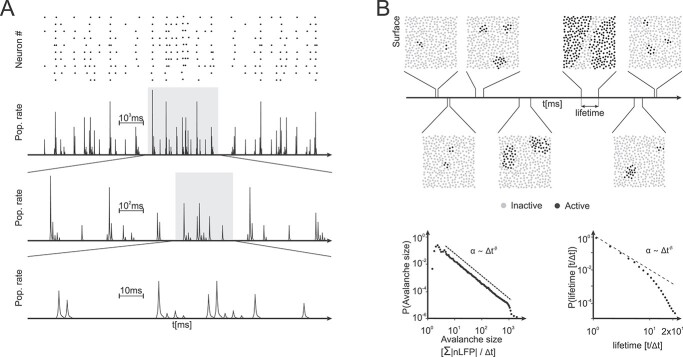
A) Schematic illustration of spike avalanches in a network of neurons. Synchronous network bursts manifest at multiple different scales, having similar statistical properties (peaks of population firing rate in time). Note that while time is zoomed in (top-down direction) the magnitude of network bursts is also scaled, with fewer neurons participating in the bursts on shorter timescales (lower peaks of the population firing rate), B) The phenomenon is conceptually illustrated in spacetime representation (top) where snapshots of avalanches with different avalanche size and lifetime (snapshot width) are visualized with relative distances representing the quiescence time. The two log–log probability distribution plots are strictly illustrative and intended to highlight rough approximates of reported observations in existing work supporting the scale-freeness of the avalanches size (left-bottom) and lifetime (right-bottom). The fitted straight lines indicate how both the avalanche size and normalized lifetime approximate a power law with the slopes given as a function of the temporal resolution $\Delta t$ and scaling exponent $\beta$. If a process within the mean-field directed percolation universality class is considered, the slopes are equivalent to −3/2 for avalanche size, respectively −2 for lifetime. The two log–log plots are given strictly for a general intuitive visualization inspired from existing work and are not a result of data analysis. The figure is inspired by simulations of recurrent microcircuits in Mureşan and Savin (2007).

Being a critical phenomenon, avalanches bear the signatures of scale-free dynamics. First, the spatial extent (size) to which avalanches propagate follows a power law with a slope close to $\alpha =-3/2$ (Beggs and Plenz 2003; Plenz 2014). This distribution is markedly different from an exponential distribution expected when avalanches do not occur by interaction between local groups of different sizes (Klaus et al. 2011). Moreover, the lifetime of avalanches likewise follows a scale-free distribution with a slope of about −2 (Beggs and Plenz 2003). Sizes of the avalanches show strong correlation with the quiescence times before and after avalanche events: The size of the following avalanche relative to its predecessor increases or decreases directly proportional to the current quiescence time (Lombardi et al. 2016). For a better intuition about these properties, Fig. 4 schematically illustrates them in a temporal and spatial manifestation. Importantly, the figure does not depict nLFP events, but network firing bursts, as those observed in simulations of excitable, recurrent microcircuits (Mureşan and Savin 2007).

Second, according to branching theory (Harris 1963), for avalanches the branching parameter $\sigma$ describing the ratio between the number of processing units in two adjacent time steps must be equal to 1 such that on average the activity is stable and does not explode or die out (Beggs and Plenz 2003), meaning that the size of the group that initiates the avalanche must be maintained throughout the avalanche (Plenz 2014). This branching value has been observed experimentally in organotypic mammalian cultures (Plenz 2012) and in humans (Shriki and Plenz 2014). For $\sigma =1$, the spatial span (size) of resulting avalanches is a power law with $\alpha =-3/2$. Both $\alpha =-3/2$ and $\sigma =1$ are necessary to demonstrate critical behavior exhibiting avalanches (Beggs and Plenz 2003; Plenz 2014).

Third, another signature of avalanches is the insensitivity of their scale-free properties to the choice of nLFP threshold used to define avalanches (Plenz 2012), as long as the threshold is above the noise limit (Beggs and Plenz 2003; Petermann et al. 2009). Lower amplitude nLFPs integrate into existing avalanches as the threshold is reduced while maintaining the same scale-free characteristics (Plenz 2014).

Fourth, avalanches span subsequent avalanches with smaller amplitudes. These sequences of avalanches are themselves organized as avalanches with decreased amplitudes of nLFPs in a scale-free and fractal manner (Petermann et al. 2009). The probability of an avalanche occurring after another avalanche of a given size has a similar relation to the Omory–Usu law that describes the decay rate and number of aftershocks following earthquakes (Osorio et al. 2010). In fact, neuronal avalanches have a lot in common with earthquake dynamics (Plenz 2014). In electrophysiology of cortical slices, it has been observed that the number of after avalanches following an avalanche of a given size is proportional to an exponential function with exponent 0.8 (Plenz 2012).

#### Factors that support SOC

A key factor that supports avalanches in particular, and SOC in general, is the balance between excitation and inhibition that avalanches are extremely sensitive to Plenz (2012). This balance seems to be precisely maintained in layers 2/3 where, during development, nested oscillations (4–100 Hz) and avalanches depend on GABA and NMDA receptors (Gireesh and Plenz 2008). Although most of the work on avalanches has been on organotypic cultures, SOC has been shown to also emerge in rat dissociated cultures during development (Yada et al. 2017). In a combined in vitro/in silico study on maturation of cortical circuits, the same staged development was observed: From an initial low activity, cultures evolve to a supercritical phase with increased activity, followed by a subcritical phase; finally they stabilize in a critical state (Tetzlaff et al. 2010). Inhibition is recognized as crucial in shaping the network during development and supporting criticality.

Plasticity mechanisms such as the spike-timing-dependent plasticity (STDP) and homeostatic plasticity were also reported to play an important supportive role for SOC as they can produce critical points within the landscape of possible states and maintain the system at criticality (Levina et al. 2007; Plenz and Niebur 2014; Zeraati et al. 2021). In fact, by adding more and more biologically plausible details, even in simple models, the critical points become large zones and the system dynamics is closer to the criticality observed in vivo (Levina et al. 2014). Perhaps more generally, dynamical synapses with limited but sufficient neurotransmitters contribute to a robust SOC behavior (Levina et al. 2007).

Another interesting question pertains to the effect of connectivity on SOC. Functional connectivity seems to be relevant for SOC, as avalanches have been shown to selectively propagate within the network along those pathways associated with functional connectivity (Moretti and Muñoz 2013; Zhigalov et al. 2017). Physical connections, on the other hand, are less essential. Multiple network structures can support SOC dynamics, including the random and fully connected networks, but only if enhanced by plasticity mechanisms (Heiney et al. 2021). Interestingly, in a recent study (Plenz et al. 2021) it has been shown that the layered structure is necessary to support complex SOC dynamics observed in vivo. Propagation times along the network connections also seem important for SOC (Khoshkhou and Montakhab 2019).

Recently, another type of criticality, namely edge-of-chaos criticality, was identified in massively parallel spike recordings from awake macaque motor cortex (Dahmen et al. 2019). This type of criticality is supported by heterogenous connections and is characterized by dynamic changes from regular to chaotic activity, despite weak pair-wise correlations among cell pairs accompanied by weak and fast fluctuations of the population activity with slowly decaying autocorrelations. The implication of this regime is that weak stimuli fed into the network are just sufficient to drive the network over the edge of stability, impacting the recurrent dynamics (Savin et al. 2006). Moreover, this criticality type provides a rich spectrum of transformations on the input stimuli, a hallmark of reservoir computing (Lazar et al. 2009).

#### Criticism of SOC

In parallel with the literature supporting the SOC hypothesis, a reasonable and thought-provoking body of criticism followed. A first argument is that the power-law-governed avalanches are not present exclusively in SOC systems. They can also emerge as byproducts in scale-free/non scale-free and hierarchical networks that operate in a sub-critical regime (Faqeeh et al. 2019). Some examples are models based on Directed/Undirected/Input–Output Correlated Directed Networks analyzed by percolation and branching process theory. Hereby, it is emphasized that SOC is mostly related to dynamics rather than connectivity (Faqeeh et al. 2019). Hence, besides quantification of power laws and branching parameters in avalanches, more analytical tools are required to support the SOC hypothesis (Martinello et al. 2017; Touboul and Destexhe 2017; Priesemann and Shriki 2018; Faqeeh et al. 2019; Wilting and Priesemann 2019; Destexhe and Touboul 2021; Heiney et al. 2021).

Other studies support this criticism by showing that apparent criticality can occur in systems far from SOC. For example, external drive with inhomogeneous Poisson processes can generate activity that displays imperfect power laws, which match experimental data for several orders of magnitude up to the cutoff point. In such cases apparent criticality can be differentiated from true criticality based on the variation of temporal scale or bin size, i.e. time window analysis (Priesemann and Shriki 2018). In another study, avalanches governed by power laws were investigated from the perspective of neutral theory, which imposes models with no free parameters that are driven by pure noise but exhibit diffuse and scale-free dynamics (Martinello et al. 2017). They also show that scale-free like avalanches can emerge in modes far from criticality. Analytical studies of sparsely connected leaky-integrate-and-fire neurons (LIF) have shown a rich repertoire of states including a self-sustained irregular regime and states in which the system oscillates but the individual cells do not (Brunel 2000). The so-called synchronous irregularity (SI) state, a far from equilibrium state in which activity is interspersed by periods of silence, can produce avalanches with quiet times consistent with the actual characteristics of the avalanches observed in vitro. Moreover, entropy shows maximal information capacity in the SI state which proves that maximum entropy is not exclusive to critical systems. Two other noncritical models manage to pass all well-established methodologies for SOC hypothesis testing, adding to the evidence that SOC testing in experimental data is not straightforward (Touboul and Destexhe 2017; Destexhe and Touboul 2021). The research of Neto et al. (2020) attempts to explain the motif of divergent results of the last two decades on this topic. The sampling technique drastically impacts the properties of the data, as coarse sampling techniques of neural activity are argued to be prone to erroneously produce power-law characteristics, whereas spike recording is a much more preferred technique to mitigate such errors and is reported to identify a reverberating (i.e. system’s activity reverberates up to hundreds of milliseconds) rather than critical regime in recorded activity.

Inherently, recorded neuronal data are limited by the subsampling imposed by the spatial grid of recording electrodes. In fact, this problem has been recognized by Plenz, and the first step in defining the cascades and avalanches, the temporal binning of nLFPs, is akin a low pass filtering necessary to avoid aliasing due to spatial subsampling. The temporal bin width is chosen to match the spacing of electrode sites (Beggs and Plenz 2003; Plenz 2014). In an effort to overcome the limited methodologies and known issues related to the quantification of power-law avalanches in subsampled data, Wilting and Priesemann have introduced a subsampling invariant estimator based on a process of first-order autoregressive representation (PAR) that quantifies how far from criticality a system is (Wilting and Priesemann 2018). PAR investigation of in vivo monkey, rat, and anesthetized cat spiking data suggest that the cortical dynamics is not in a critical state but rather in a reverberating regime, sufficient to integrate information. In contrast, another proposed metric, the “Deviation from Criticality Coefficient,” shows that in vivo activity in the visual cortex is robustly organized in the proximity of criticality and that perturbations push the visual system away from the critical state (Ma et al. 2019).

#### Relevance of input

In several studies on criticality, networks are investigated that are driven by input, usually with Poisson statistics. It can be argued that systems at equilibrium, not subjected to external stimuli, require criticality to maintain stability but that when input is present, such systems require a branching parameter that is lower than 1 and cannot attain criticality under the condition of external drive (Williams-García et al. 2014). However, when investigating models of brain dynamics, we would like to challenge the community to consider systems that exhibit endogenous dynamics and are not driven by external stimuli, but their ongoing dynamics is merely perturbed by such stimuli (Mureşan and Savin 2007). Indeed, it has been long argued that cortex is actually not driven but perturbed by external stimuli (Arieli et al. 1996) and there is evidence that thalamic input to cortex is very weak (Bruno and Sakmann 2006). Robust onset responses to sensory inputs (Albrecht et al. 2002; Frazor et al. 2004; Savin et al. 2006) may actually occur because the cortex is in a critical, ready-to-burst regime, where tiny perturbations are transformed into robust network responses (for more details, see Section Functional Role of Fractal Structure and Scale-Invariant Dynamics). Establishing how much of this mechanism is explained by the criticality or the reverberation hypothesis remains a challenge for future studies.

#### Scale-free properties of neural coding

Another important question is related to the dimensionality of the neural code at population level (Cunningham and Yu 2014). A recent study (Stringer et al. 2019) used simultaneous recording of > 10,000 neurons and evaluated the geometry of the (average rate) encoding space in visual cortex during presentation of flashed natural images. They found that the variance explained by stimulus-related principal components decays as a power law, with exponent close to 1. Interestingly, this seems to be a property endogenous to visual circuits as it does not depend necessarily on the spatial statistics of input images. Importantly, the power-law scaling of explained variance suggests that, at least when it comes to the average firing rate of neural populations in response to flashed inputs, the neural code behaves as a smooth manifold, in between nonredundant, efficient coding, and robust but partially redundant representations. However, such analyses of average firing rates of neural populations ignore the dynamical nature of neural activity as trajectories in high-dimensional space perturbed by time-varying inputs (Jurjuţ et al. 2011). In the future it will be interesting to expand the approach introduced by Stringer et al. (2019) to the case of dynamical representations of more realistic, time-varying stimuli (Maldonado and Babul 2007; Maldonado et al. 2008). In addition, another challenging direction will be to establish the relevance of SOC or reverberation mechanisms for enabling brain circuits to encode information in a way that is both efficient and robust.

### Scale-free dynamics at the macroscale

Historically, scale-free brain dynamics has been identified in studies evaluating the meso- and macroscale properties of brain activity from EEG. One of the pioneers of the field was Walter J. Freeman whose work has initially focused on the chaotic nature of EEG, recorded in olfactory stimulation paradigms (Skarda and Freeman 1987), and later gradually shifted to the properties of brain oscillations in humans. In several studies, Freeman and colleagues demonstrated that human EEG obeys a $1/f$ property (Freeman et al. 2000, 2003). Freeman argues for the dynamical, scale-free organization at the mesoscopic level of local circuits, which leads to macroscale coordination that in turn feeds back as global modulation of the local domains (Freeman 2005). The $1/f$ property of brain signals’ spectra has been equated to signs of SOC and scale-invariance (He et al. 2010; Werner 2010; He 2014). Indeed, LRTCs with robust power-law scaling behavior were also found in spontaneous oscillations from MEG and EEG (Linkenkaer-Hansen et al. 2001).

More recent studies investigated how different cognitive, emotional, and conscious states change scale-free dynamics at the macroscale. In the resting state, the spatial distribution of activity indicates that the overall system rests at the critical point of a second-order phase transition (Tagliazucchi et al. 2012). Furthermore, the phase difference between electrodes that are placed across the brain exhibits a $1/f$ distribution (Thatcher et al. 2009). However, depending on the task, the scale-invariant dynamics changes. Cognitive effort seems to decrease fractal-scaling (Churchill et al. 2016) and increase multifractality (La Rocca et al. 2018), suggesting the differential engagement of local versus global circuits by the task. Emotions impact the fractal state of the brain as well, with humor eliciting more and fear leading to less scale-free like regime (Ruiz-Padial and Ibáñez-Molina 2018). Finally, the scale-free, critical dynamics of the brain is strongly suppressed in states of low consciousness. Sleep decreases $1/f$ properties of the signal, as well as of the distribution of activity over the brain, by suppressing fine spatio-temporal patterns (Pereda et al. 1998; Shen et al. 2003; Lee et al. 2004; Freeman et al. 2006; Weiss et al. 2009, 2011; Miskovic et al. 2019). Likewise, as the brain enters anesthesia, the fractal-scaling exponent increases (Gifani et al. 2007), indicating that the temporal correlations in the data diminish.

The implication of SOC and scale-free activity is also evidenced by the impact that epilepsy and development have on these properties of the activity. Berthouze et al. showed that during development from newborn to 55 years, power-law statistics are prevalent in all frequency bands, and that the dominance of this type of activity changes with age (Berthouze et al. 2010). This finding has been replicated in Namazi and Jafari (2018). Epilepsy has been shown to impair SOC in studies on rats (Zhou et al. 2007) and humans (Meisel et al. 2012). Meisel et al. proposed that seizures are caused by a failure of adaptive SOC and showed that during sustained wakefulness the LRTCs diminish, suggesting that criticality itself is lowered (Meisel et al. 2017). These results suggest that SOC may be one of the principal modes, which underlie normal brain activity.

### Scale-free properties of motor control and behavior

Scale-free properties do not only characterize the dynamics of various neural subsystems but have also been shown to be expressed at the level of different control systems and behavior—perhaps a powerful explanation for why nervous systems must display fractal structure and scale-free dynamics. Indeed, many organisms must cope with an environment that spans scales covering multiple orders of magnitude, in both space and time. For example, in ecology it has been shown that ecosystem dynamics obeys power-law scaling (Cobain et al. 2019): The variance in population abundance scales as a power law of the mean (Taylor 1961). Another famous example is the species-area curve (Harte et al. 2009; O’Dwyer 2015), whereby the number of species scales as a power law of the area of the habitat. Similarly, along the temporal dimension, the evolutionary distance scales as a power-law function of species number (O’Dwyer 2015).

To start the discussion about scale-free properties in the motor system, it is useful to first note that scale-invariance is already manifest in the statistics of miniature end-plate potentials at the neuromuscular junction (Takeda et al. 1999; da Silva et al. 2020). Such phenomena are then mirrored in the statistics of several classes of behavior. In humans, the stride-to-stride variability, also called gait dynamics, exhibits nonrandom statistics (Hausdorff et al. 1995; Hausdorff 2007). Similar findings were reported for human running (Jordan et al. 2006) and as a function of various walking speeds (Jordan et al. 2007). Furthermore, it was demonstrated that similar scale-free properties can be found in the human respiratory dynamics, and that such fractal properties degrade with age or under the influence of various diseases (Peng et al. 2002).

In humans, forearm movement during wakefulness obeys scale-free statistics (Hu et al. 2004). This property does not depend on the particular subject and is not affected by the change in average activity level. In a follow-up study, it was reported that scale-free statistics are shared also by rat locomotion, spanning 24 h (Hu et al. 2007). Indeed, not only locomotion or active movement are regulated in a scale-free manner but also heart rate (Hu et al. 2008), which shows power-law behavior under the control of the suprachiasmic nucleus (SCN; Pittman-Polletta et al. 2013). Importantly, the SCN seems to be involved in regulating scale-free behavior at the large-scales, beyond 2–4 h (Hu et al. 2007). Lesioning or ablating the SCN in rats leads to a degradation of fractal properties in behavior and heart rate, suggesting that the SCN is a major player in the regulation of scale-free dynamical patterns at large timescales (Pittman-Polletta et al. 2013). These studies already hint at the relationship between scale-free properties of behavior and heart rate, and certain brain structures that may be involved in the regulation of such properties.

A wide body of evidence has accumulated that indicates fractal-like scaling properties of human behavior. In 2001, David Gilden proposed that $1/f$ noise is actually the signature of intrinsic dynamics associated with the formation of representations (Gilden 2001). He showed that such “noise” is manifest in many important psychophysical measurement paradigms, such as speeded judgment, discrimination accuracy, and production. Along the same lines, Kello and colleagues reviewed scaling laws observed in various setups in cognitive sciences, pertaining to perception, action, memory, language, or computation (Kello et al. 2010). Importantly, $1/f$ scaling seems to be ubiquitous in all these systems (He 2014), with the observation that the scaling exponents can vary across individuals, tasks, or time (Kello et al. 2010; Ravi et al. 2020).

A more recently addressed question tackles the way scale-free properties of behavior are also mirrored and caused by brain dynamics with similar statistics. It was shown that brain processes like the infra-slow oscillations can modulate behavioral performance. The phase but not the amplitude of infra-slow (0.01–0.1 Hz) fluctuations reflected in the EEG of the ongoing brain activity is correlated with subjects’ ability to detect a sensory target in a somatosensory detection task (Monto et al. 2008). This and other evidence (VanRullen et al. 2006) suggest that statistics of brain dynamics, motor output and behavior closely follow each other. Indeed, a recent study demonstrated that timing-error sequences performed by humans obey scaling properties inducing long-range correlations that are also simultaneously found in the amplitude modulation of multiple resting-state brain oscillations. Furthermore, the scaling exponents characterizing errors during taping behavior are predicted by fluctuations in precentral alpha oscillations (9–13 Hz; Smit et al. 2013). Going one step further, Palva and colleagues studied the relationship between LRTCs evidenced by power laws in the fluctuation of neural oscillations, in the properties of neural avalanches, and in the behavioral performance fluctuations (Palva et al. 2013). They showed that scaling laws of oscillation fluctuations and neural avalanches are strongly correlated with each other and, in addition, correlated to the scaling laws of behavior. Taken together, these recent advances demonstrate that scale-free properties of behavior are tightly related to the scale-free dynamics of brain circuits.

## Functional role of fractal structure and scale-invariant dynamics

The previous sections reviewed existing literature on scale-invariance, fractality, criticality, and power-law distributions in natural systems, in the structure of the brain, and in brain dynamics. Following this evidence and going beyond the pure observational perspective, this section attempts to distill from the vast body of literature presented some reasons why these properties might be important for brain function. The central questions are the following: If fractal structure and scale-free dynamics are so ubiquitous in the brain, what role do they subserve? Are they fundamental features supporting mechanisms for perception and cognition? Why are they so prominently expressed in the neocortex, the ultimate computational machine?

Specific features of fractal systems have been analyzed across multiple scientific fields. This immediately suggests that drawing parallels between, e.g. physical systems at the critical point of a phase transition and the brain, can be helpful. However, we must bear in mind that in contrast to many physical systems, the fundamental microscopic principles behind the observed scale-invariance in the brain are largely unknown. Physical laws mediating interaction between constituents of matter are well understood, whereas general models for brain structure and dynamics mostly have not been created from first principles but are usually constructed ad-hoc, to fit selected experimental observations. Consequently, while seemingly modeling the data, they do not explain them. Thus, generalizing properties from specific models is often difficult, sometimes speculative.

In the following, we enumerate and discuss relevant properties and capabilities of fractal, scale-free, critical, and power-law distributed systems.

### Criticality is useful for robust and fast reaction to sensory inputs

One of the most prominent features of neocortical circuits is their ability to respond vigorously to changes in sensory evidence. In the visual system, such strong and fast modulations of ongoing activity, triggered by changing visual stimuli, are called “onset” (when the stimulus appears) and “offset” (when the stimulus disappears) “responses” (Albrecht et al. 2002; Frazor et al. 2004; Nikolić et al. 2007). The prominence of both onset and offset responses indicates that the thalamo-cortical circuitry acts more like a differentiator than an integrator (Savin et al. 2006). This is essential for the ability of an organism to react rapidly to dangerous changes in its environment. From an evolutionary perspective, such mechanisms may have evolved to endow brains with the ability to efficiently process sensory inputs and enable fast reactions, while also inferring causality (Friston 2005). However, these may also underlie a more subtle, general dynamical principle that is rooted in criticality and governs the fundamental-processing principles in the brain.

To date, it is still not fully understood how onset or offset responses are generated by cortex. One possibility is that, engaged by various sensory modalities, the thalamus exerts a strong drive of layer 4 cortical neurons, consequently engaging the entire cortical circuit. However, this is contradicted by experimental evidence. It was shown that thalamocortical axons form only weak inputs to cortex, accounting for at most 15% of synaptic connectivity (Bruno and Sakmann 2006). In addition, cortico-thalamic feedback connections vastly outnumber the feedforward fibers towards cortex by a factor of 10 to 1 (Crandall et al. 2015). This strong recurrence is also shared by the cortex, whereby both feedforward and feedback projections are prominent (Berezovskii et al. 2011; Mejias et al. 2016). It is thus evident that vigorous circuit responses are not generated by strong input drive but by some other mechanisms, which ensure that small perturbations are transformed into robust responses. Indeed, it has long been suggested that, rather than being driven by inputs, the cortex processes perturbations (Arieli et al. 1996).

Mechanistically, a plausible scenario is that weak perturbations from sensory inputs unleash strong excursions of the dynamical trajectories of cortical circuits, which are in a “ready-to-burst,” critical state. It has been shown that in isolated cortical slabs, spontaneous circuit bursting emerges in the absence of any thalamic input and stabilizes as the size of the slab is increased (Timofeev et al. 2000). One possibility is that such criticality in cortical circuits emerges via an interplay of reentrant excitatory loops, effectively generating positive feedback, and other mechanisms that stabilize network dynamics and prevent it from becoming epileptic. This is consistent with the well-known recurrence of cortical circuits (Thomson and Bannister 2003; Douglas and Martin 2004). It has previously been shown that recurrent positive feedback coupled with integrative properties of composing neurons is able to reproduce onset and offset responses (Savin et al. 2006), as well as vigorous spontaneous, avalanche-like bursting triggered by spontaneous miniature synaptic potentials (Mureşan and Savin 2007). Ready-to-burst, critical circuits also need some mechanisms for dynamical stability that prevents activity runaway. This may be ensured by the balance of excitation and inhibition (Tiesinga and Sejnowski 2009), by synaptic delays, or resonant membrane properties (Mureşan and Savin 2007; Moca et al. 2014).

Importantly, critical cortical circuits are able to vigorously merge perturbations induced by sensory changes with their ongoing dynamical context (Arieli et al. 1996; Ahissar and Arieli 2001; Bertschinger and Natschläger 2004; VanRullen et al. 2006). At functional level, this has several useful consequences. First, responding only to changes in sensory evidence enables efficiency in using computational resources. Second, it has been suggested that networks in a critical regime are better suited to perform complex computations on time series (Langton 1990; Bertschinger and Natschläger 2004; Legenstein and Maass 2007a; Boedecker et al. 2012). Finally, the robust response of cortical circuits to small sensory perturbations may also ensure a normalization that decouples the magnitude of the response from the magnitude of the input, dramatically increasing signal-to-noise ratio (Shirai et al. 2020).

### Critical and scale-invariant systems optimize information theoretic and algorithmic properties

Although scale-invariance and criticality are distinct concepts, they are strongly coupled and aim to explain the underlying mechanisms and related effects arising from the spatial, topological, and dynamical characteristics of a broad spectrum of both equilibrium and nonequilibrium complex systems. A comprehensive view is given in Khaluf et al. (2017), presenting scale-invariance as a general property that a system can exhibit. According to Khaluf et al., real-world complex systems are thought to intrinsically exploit scale-invariance as a form of optimization, to ensure functionality, adaptability, and robustness in a limited, yet broad range of scales. Coherent, efficient, and rapid stimulus-based adaptability can be achieved by global information propagation through a scale-invariant interaction of the system’s subunits in the brain network. Regarding robustness, scale-free systems are particularly useful given their ability to conserve the system’s integrity and the average characteristics when facing random failures (Barabási and Bonabeau 2003; Khaluf et al. 2017). Studies have shown that scale-free systems maintain functionality even after a failure rate of 99% of the node population, given the chance that the hubs remain intact (Cohen et al. 2000).

There are also systems that explicitly show the benefit of operating in the vicinity of a continuous phase transition. Langton examined mutual information between cells of cellular automata at adjacent points in time as well as the entropy of the system, finding that there is some optimal entropy where cellular automata display large spatial and temporal correlations (Langton 1990). Similar results were evidenced in more complex systems, like neural networks (Kauffman 1993), suggesting that it is beneficial for a system to stay close to criticality in order to exhibit interesting information-processing capabilities (Bertschinger and Natschläger 2004).

Inspired by the evolutionary perspective, Carteret et al. investigated the thermodynamic implications and benefits of systems thriving close to criticality (Carteret et al. 2008). Results indicate that networks operating close to criticality can maximize not only energy efficiency but also pair-wise mutual information (peaking slightly in the subcritical regime). The latter is a powerful indicator to quantify coordination efficiency between multiple elements of a network (Carteret et al. 2008; Ribeiro et al. 2008). In addition, analysis of energy efficiency, network bias, and network sensitivity reveals that the power efficiency remains maximal as the network is maintained in the proximity of criticality (i.e. sensitivity = 1), regardless of the bias value, indicating that such a state is robust to variability of other network-related parameters. Similar observations were made in Lazar et al. (2009), where self-organized recurrent networks with excitatory-inhibitory neurons and plasticity mechanisms were analyzed by contrast to static random reservoirs. Additional research regarding the properties of random Boolean networks operating in a critical regime can be found in Nykter et al. (2008). However, a more recent study, investigating a plastic spiking network on a neuromorphic chip suggests that systems do not necessarily benefit from criticality for any task: Although network capacity is maximal at criticality, this may only benefit complex but not simple tasks (Cramer et al. 2020).

Tomen and Ernst focused on the impact of the critical or near-critical dynamics of a system on visual information-processing performance (Tomen and Ernst 2019). For figure-ground segregation, their model benefits from criticality manifest in subnetworks, offering the best results in a synchrony coding scenario using a limited number of neurons. A second scenario suggests that, as the model operates closer to the transition boundary, its ability to rapidly enhance stimulus representation with minimal modulation of input gain increases significantly, leading to flexible tuning of information processing by attentional mechanisms. Moreover, task performance is argued to be optimal at a state of proximal criticality, where selective attentional mechanisms regulate oscillatory activity in local circuits. The highest entropy arises at the boundary between the subcritical and critical phases, with a maximum in a region that is slightly subcritical. This level drastically decreases as the model shifts towards supercriticality (Tomen and Ernst 2019). These conclusions are enforced by previous work (Tomen et al. 2014), emphasizing the importance of the coupling emerging from enhanced gamma-oscillations, when the model is close to criticality. Similarly, Gautam et al. (2015) showed that in somatosensory cortex the critical state is beneficial, whereas both subcritical and supercritical states result in a low dynamic range.

Several authors emphasize the importance of criticality and edge-of-chaos computation for online, real-time stimulus processing in complex dynamic systems (Bertschinger and Natschläger 2004; Legenstein and Maass 2007a, 2007b). A highlighted aspect is the relation between scale-invariant structure/topology and scale-invariant dynamics. Structure and topology are vital to ensure scale-invariant dynamics, which in turn are essential for online, real-time computation. In addition, a quantitative metric such as the linear separation property was proposed for the empirical evaluation of the computational power of kernel-quality of complex systems, such as neural microcircuits, showing that highest performance is obtained at the edge-of-chaos.

Similarly, echo state networks (Jaeger and Haas 2004) with feedback connectivity present the ability to perform online computation on stochastic stimuli, like their biological counterpart (Boedecker et al. 2012). To evaluate the importance of criticality in echo state networks, Shannon entropy, joint and conditional entropy, mutual information, short-term memory capacity, information storage, and information transfer were computed while varying the order parameter of the network. Measurements indicate maximization of these features as the order parameter approaches the critical value. Nonetheless, it is argued that not all tasks can be solved optimally by systems operating at criticality, and maximization of such metrics does not guarantee development of complex dynamics. This may be an argument explaining why some complex systems do not develop naturally to operate at the edge-of-chaos (Boedecker et al. 2012).

### LRTCs may be useful for memory mechanisms

One of the earliest studies to interpret correlations as a proxy for memory is (Langton 1990), where periodic patterns emerging in cellular automata were considered as signs of memory. Since then, the general view is that LRTCs are maximized at criticality (Lazar et al. 2009; Wilting et al. 2018; Wilting and Priesemann 2018; Hochstetter et al. 2021) and are associated with the brain’s ability to adjust the responsiveness to different tasks, contributing to memory, integration of information, and increased signal-to-noise ratio (Shirai et al. 2020).

The ability to integrate information over time may be supported by LRTCs during decision-making and working memory. There is now sufficient experimental evidence that LRTCs occur in the brain (Palva et al. 2013; Meisel et al. 2017; Plenz et al. 2021), and concepts of short-term and working memory have been extensively studied, especially in the context of goal directed tasks (Chai et al. 2018). Under sleep deprivation, LRTCs are reduced concomitantly with diminished brain function (Meisel et al. 2017). Like avalanches, LRTCs are associated with critical dynamics (Poil et al. 2012; Plenz et al. 2021), and may be present in local circuits even under anesthesia. Indeed, cortical responses of anesthetized cats, induced by one stimulus, can persist even after a new stimulus has been presented, such that both representations coexist and interact (Nikolić et al. 2007).

In self-organized recurrent neural networks (SORN; Lazar et al. 2009), plasticity mechanisms are necessary in order to attain a critical state, but after it is achieved, they are no longer necessary to maintain it (Del Papa et al. 2017). More interestingly, although random input does not perturb the SORN’s critical state, structured input seems to cause it to break down. In a similar vein, there are numerous studies on criticality and its relation to synaptic plasticity, like spike-time-dependent plasticity (STDP). For example, sequences can be memorized via STDP while criticality is maintained (Scarpetta et al. 2013, 2018; Scarpetta and de Candia 2014). In addition, Hebbian plasticity and STDP both account for different aspects of learning: Although STDP fosters the formation of sequence memory, Hebbian plasticity forms associations (Zeraati et al. 2021), both of which have been shown to occur under critical states (de Arcangelis et al. 2006).

While LRTCs may be optimal for reservoir computing and working-memory tasks, criticality is not always the optimal regime (Shirai et al. 2020). A reservoir endowed with homeostatic and plasticity mechanisms, operating in a subcritical regime, outperforms static networks because STDP can incorporate the temporal structure of the task into the structure of the network (Lazar et al. 2009). Because the structure of the task is incorporated within the network itself, the optimal regime for that particular task may not lay at criticality. Rather, criticality is postulated to optimize information processing in general, rather than for a specialized task. Although LRTCs could optimize complex tasks that require integration over long periods of time, the simple ones are solved more efficiently in a subcritical regime and could even be hindered by critical dynamics (Cramer et al. 2020).

Intuitively, reverberating, irrelevant activity that fades too slowly may hamper the ongoing processing. Recent experimental data from behaving monkeys, anesthetized cat visual cortex, and rat hippocampus suggest that the brain is rather in a subcritical regime (Wilting and Priesemann 2018). One possibility is that criticality may support generic computations, while specializing circuits for a specific purpose could render them subcritical. Then, the question is how critical and subcritical regimes reconcile in the brain. It was proposed that circuits could transition quickly between different nuances of critical and subcritical states, one of these subcritical states being a reverberating regime that implements dynamic adaptive computation (Wilting et al.^2018^). In this regime the brain could switch continuously along a spectrum of various responses to minimal perturbations that lay between 2 extrema: First, a balanced state, in which spiking characteristics are asynchronous and irregular, but which is optimal for fast reaction to stimuli, and second a critical state, characterized by long-range spatial and temporal correlations suitable for integration (Wilting and Priesemann 2018, 2019).

### Scale-free dynamics may be optimal for processing of inputs with 1/*f* properties

**Fig. 5:**
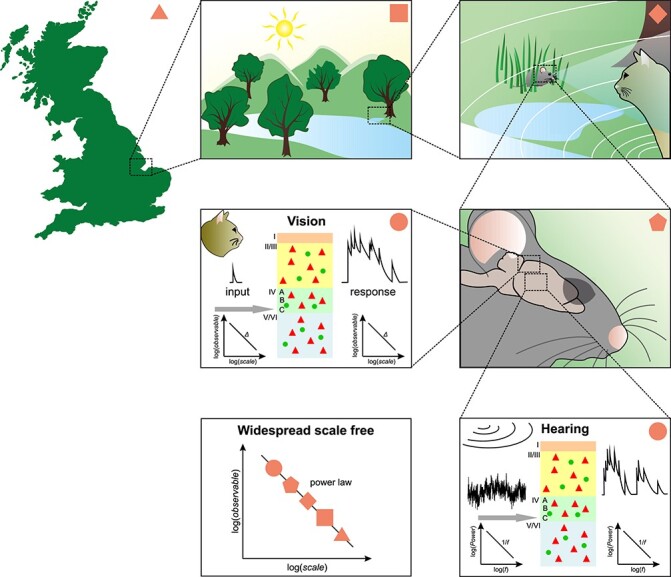
Fractal structure and scale-free dynamics at different spatial and temporal scales. The coast of Britain is a notable example for statistical self-similarity with scales of 1–100 km. On a smaller scale of 100 m—1 km, ecological environments also display many cases of self-similarity (e.g. fractal branching of trees) as well as features shared with the larger scale (lakes vs. seas). Even smaller, the pieces of local environment also resemble the larger scales, e.g. grass patches resemble patches of trees, small ponds mirror larger lakes, etc. in this local environment, animals thrive, and their brains take the scaling process even further. For example, a mouse hiding in the grass is hearing and seeing vaguely what appears to be a predator. These weak signals with $1/f$ properties translate into vigorous responses in both visual and auditory cortex that share the same statistical properties. Finally, the mouse’s brain also exhibits self-similarity on different scales and some of its structures mirror those expressed on larger spatial scales. For example, the branching structure of dendritic trees is similar to that of trees in nature.

One of the arguments for ascribing a functional role to scale-free dynamics/fractal structure in the brain is that many perceptual signals arising in nature also have such scale-invariant/statistical self-similar properties (see Fig. 5). Indeed, to process data with certain statistical properties it may be advantageous for the system to exhibit similar properties.

As discussed before, output patterns generated by the brain have fractal/scale-invariant properties when enabling motor (e.g. wrist movement or walking) or physiological parameter control (e.g. heart rate). On the input side, there is evidence that brains prefer to process signals whose properties match their own internal structural/dynamical organization. Gilden summarized the difference between white noise spectra ($1/{f}^0$), pink noise ($1/{f}^1$), characteristic for scale-free systems, and brown noise ($1/{f}^2$), characteristic for random walk (Gilden 2001). When exemplified on sounds, as also pointed out by Gardner (1978), these different types of spectra are perceived and processed differently by the brain. On one end, white noise appears totally random, and it is difficult to listen to for long periods of time. On the other end, brown noise is too predictable and also annoying. In between, sounds that exhibit $1/f$ spectra are easy and pleasant to process by humans (Voss and Clarke 1978). Indeed, it was shown by Voss and Clarke that both music and speech exhibit such $1/f$ spectra (Voss and Clarke 1975).

Not only music and speech display interesting spectral characteristics. For natural images, it has been shown that the amplitude spectrum as a function of spatial frequency exhibits an approximately $1/f$ structure (Tolhurst et al. 1992), which means the power spectrum is distributed more like brown noise ($1/{f}^2$), reflecting strong local correlations that are spatially limited (van der Schaaf and van Hateren 1996). However, the spectral profile can depend widely on the particular image category (Torralba and Oliva 2003) and the particular environment from where the images have been sampled (Balboa and Grzywacz 2003). Importantly, however, the brain does not process static images falling onto the retina but sequences of such images, with rich spatiotemporal features that emerge from movements or active visual sampling, via saccades and fixations (Maldonado and Babul 2007; Maldonado et al. 2008). Therefore, not only the spectral characteristics of images is important, but the spectral properties of the spatiotemporal features of the dynamical visual flux being processed by the brain. Recent findings indicate that for movies with natural image characteristics, the spatiotemporal amplitude spectrum obeys on average a $1/{f}^{0.5}$ characteristic, which implies that the power spectrum has a $1/f$ distribution (Isherwood et al. 2021). These recent results suggest that the visual stream processed by the brain under naturalistic viewing conditions exhibits LRTCs. A tantalizing question that should be addressed by future studies is whether active visual sampling of a scene is performed such that the resulting spatiotemporal power spectrum gets as close as possible to $1/f$ characteristics for optimal extraction of visual information.

### Fractal systems may be useful to represent the relationship between coarse context and detailed focus

One of the most difficult problems brains must address is the necessity to relate local information to global context or relating constituent parts to their wholes. Fractal structure and scale-free dynamics could putatively resolve such relationships, both in the spatial and temporal domains.

In the spatial dimension, several topological relations are either preserved or created in cortical space, like retinotopic mapping of the visual field, or tonotopic mapping of frequencies. In both cases, the spatial/frequency information is mapped in an ordered fashion along the spatial extent of the cortex. Determining the relationship between features at various spatial locations (for the case of objects), or between amplitudes of different frequencies (for the case of sounds), is crucial for visual and auditory recognition, respectively. Connecting the spatial locations in the cortical representation via fractal structure may be useful for the establishment of relations between constituent elements of objects and sounds. Bieberich suggested that recurrent fractal networks facilitate simultaneous access to local and global context, enabling integration of multiple details across multiple scales (Bieberich 2002).

The temporal domain is as relevant as the spatial domain. Brains possess rich dynamics, processing continuously incoming sensory streams and generating continuously unfolding behaviors. Visual scenes are never perceived in a static fashion, but visual inputs vary significantly in time during naturalistic visual sampling. Even during fixations, images on the retina are not fixed but fluctuate as a result of micro nystagmus (Riggs and Tulunay 1959; Fluur 1970; Coren and Porac 1974). Furthermore, the auditory stream is by its nature dynamic, be it speech, music, or natural sounds. Here, sequences of frequency distributions unfold in time and must be integrated, and their relatedness deciphered in order to make sense of the world of sounds. In both cases, correlations over multiple temporal scales contain critical information that needs to be accessible to the brain. Scale-free dynamics may be optimal for establishing correlations and relatedness across a wide temporal domain, especially since incoming auditory and visual streams exhibit $1/f$ statistics.

We argue that spatial and dynamical fractality also enables powerful computations of part-whole relationships by using a universal processing strategy. If the same computational process is replicated across all relevant scales, the system does not need any specialized computational strategies for each level. Indeed, if one considers the evolution of nervous systems, where larger and larger circuits of neurons were aggregated, it only seems natural that the same computational principle was replicated across different scales and the underlying neural structure grew via a fractal accumulation process.

**Fig. 6:**
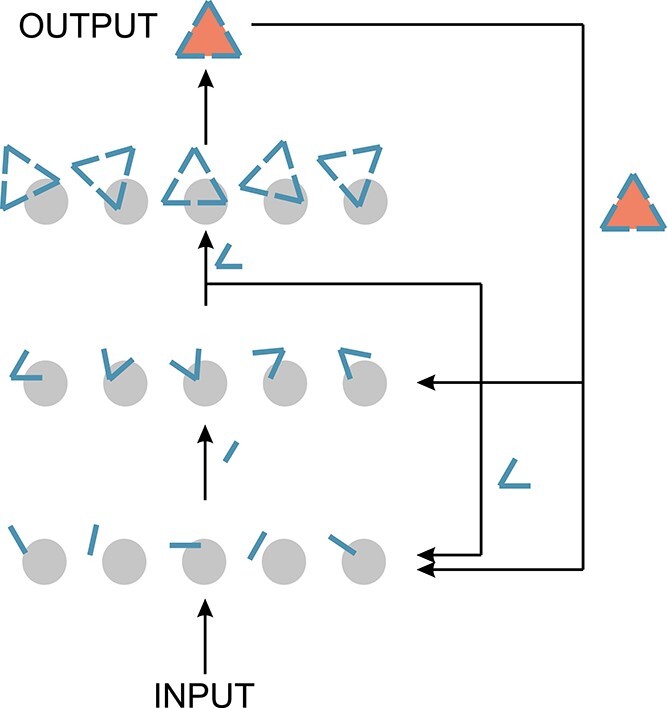
Computational principle based on recurrent fractal neural networks that integrate detailed focus with global context. Elementary features are integrated into complex representations in an ascending, convergent neural hierarchy. On the other hand, the global context from higher levels is fed back to every lower level. As a result, each processing element has access both to its local focus and to the global context.

The popular deep learning framework used today (LeCun et al. 2015) is rooted in the classical convergent hierarchical computation of neural networks (Churchland and Sejnowski 1992), which are, interestingly, fractal trees. Indeed, the same computational principle is more or less replicated at different layers, which correspond to different scales or abstraction levels as one advances up the hierarchy. Although very successful as tools for pattern recognition, deep neural networks can only encode the relation between parts and their wholes. For example, when used for object recognition, the spatial information is gradually abstracted away and lost as one advances through the network. As a result, it is not possible to compute, at the output of the network, the relation between wholes and their parts, i.e. neurons that code for different parts are “blind” to the purpose of the global computation. Bieberich has proposed that encoding of reciprocal part-whole relationships is possible via integration of local and global states performed by feeding back the global network output as dendritic input to each neuron in the network, thus forming a recurrent fractal neural network (Bieberich 2002). In this scenario, local information of individual nodes converges to compute the global output, which, however, is accessible at every local node (Fig. 6). Recurrent fractal organization may therefore be useful to render the system simultaneously “aware” of both the presence of parts and of the whole aggregating them.

## Concluding remarks

We have reviewed a wide body of evidence that self-similarity in geometrical sense (fractal geometry), in connectivity patterns (fractal networks), and in activity (scale-free dynamics) is widely expressed in the brain. From a structural point of view, fractal arborization is manifest at both the microscopic level (dendrites and axons) and at macroscopic level (long-range, inter-area connecting fibers). Although the microscopic and macroscopic structures are reasonably well mapped out, the geometry of mesoscopic connectivity is yet to be deciphered, at least in the case of mammalian neocortex. Paradoxically, from the point of view of dynamics, the mesoscopic level is probably the best understood. In cortex, the more superficial processing layers 2/3 express scale-free dynamics, exhibiting avalanche-like statistics that are a hallmark of criticality. At the macroscopic level, global activity fluctuations measured with EEG and MEG also exhibit $1/f$ spectral characteristics, indicative of LRTCs, specific to scale-free dynamical systems. From a dynamical point of view, the least understood is the microscopic level, where recent evidence accumulates that membrane potential fluctuations in individual cells also show signs of criticality.

Although not every structure in the brain displays fractality and not all dynamical patterns are scale-free, it is now an established fact that both geometric and dynamic self-similarity are vigorously expressed in the brain. Importantly, incoming sensory streams and outgoing behaviors also appear to have properties that tie them directly to scale-invariance. We argue that old and new evidence paint an increasingly clearer picture about the functional role of self-similar geometry (fractal structure) and self-similar dynamics (scale-free) in the nervous system. These are in all likelihood not mere correlates, without functional relevance, but could support certain types of computations that are fundamental to the way brains operate. In addition, the nature of sensory inputs and the constraints of the environment likely require fundamental computational principles that take advantage of self-similarity across spatial and temporal domains. Although all this evidence points into a clear direction, it is surprising that very few attempts have been made to construct useful computational models based on such principles.

To conclude, we are still arguably at the beginning of the road towards understanding the relevance of fractality and scale-invariance in the brain. While maintaining a healthy degree of skepticism, we must probe harder, both theoretically and experimentally (Muñoz 2018). Possibly, the quest for unraveling the role of fractal structure and scale-free dynamics for computation is going to bring about the next big leap in brain research and artificial intelligence.
