# Correction to: The fractal brain: scale-invariance in structure and dynamics

**DOI:** 10.1093/cercor/bhad335

**Published:** 2023-09-01

This is a correction to: George F Grosu and others, The fractal brain: scale-invariance in structure and dynamics, *Cerebral Cortex*, Volume 33, Issue 8, 15 April 2023, Pages 4574–4605, https://doi.org/10.1093/cercor/bhac363

In the originally published version of this manuscript, the last grant in the Funding section was given as `GAMMA-CXCD'. This has been corrected online to `GAMMA-CXCD code PN-III-P1-1.1-TE-2021-0709'.

